# Supplementary material for: Microbial eukaryotes have adapted to hypoxia by horizontal acquisitions of a gene involved in rhodoquinone biosynthesis
Source: eLife. 2018 Apr 26;7:e34292. doi: 10.7554/eLife.34292 (PMC5953543; doi:10.7554/eLife.34292)

Tree 1 - RQUA + UBIE + UBIE-related clades
406 sequences, 114 sites

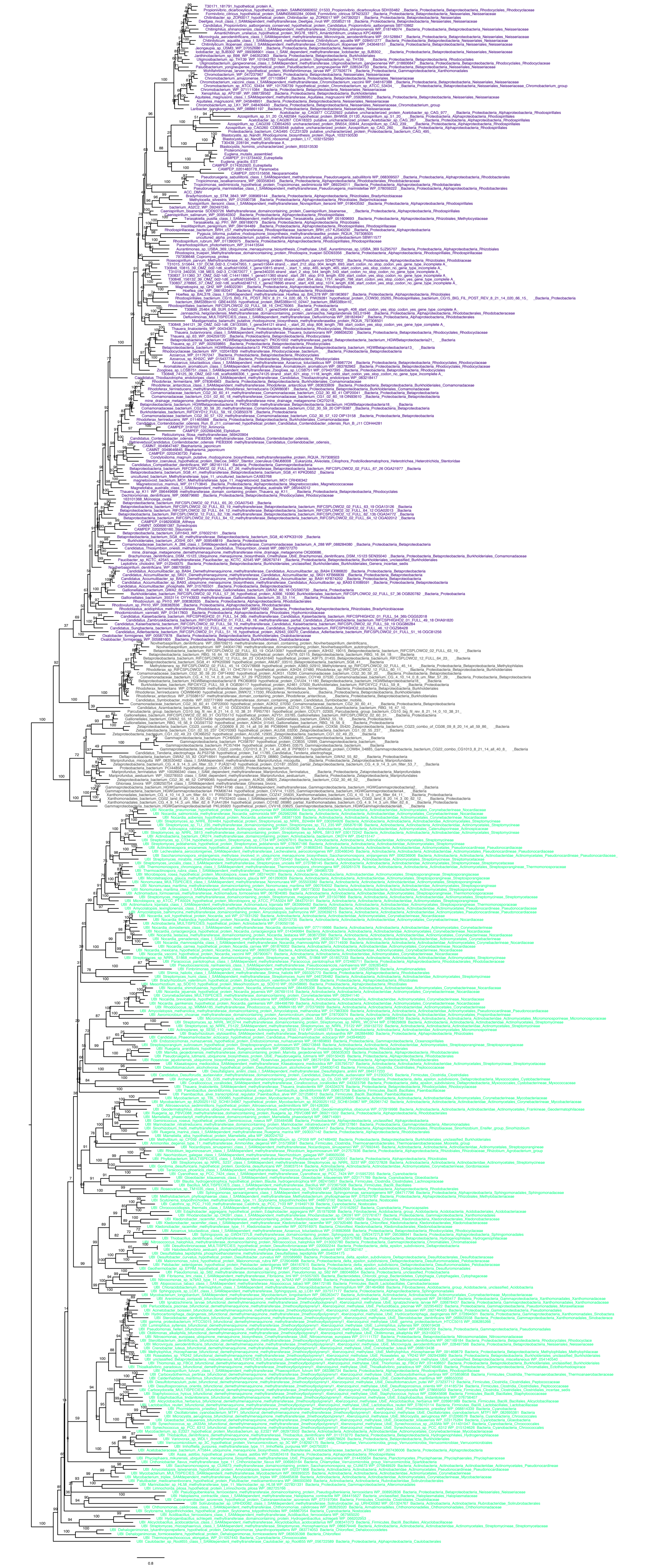

Tree 2 - RQUA Full dataset

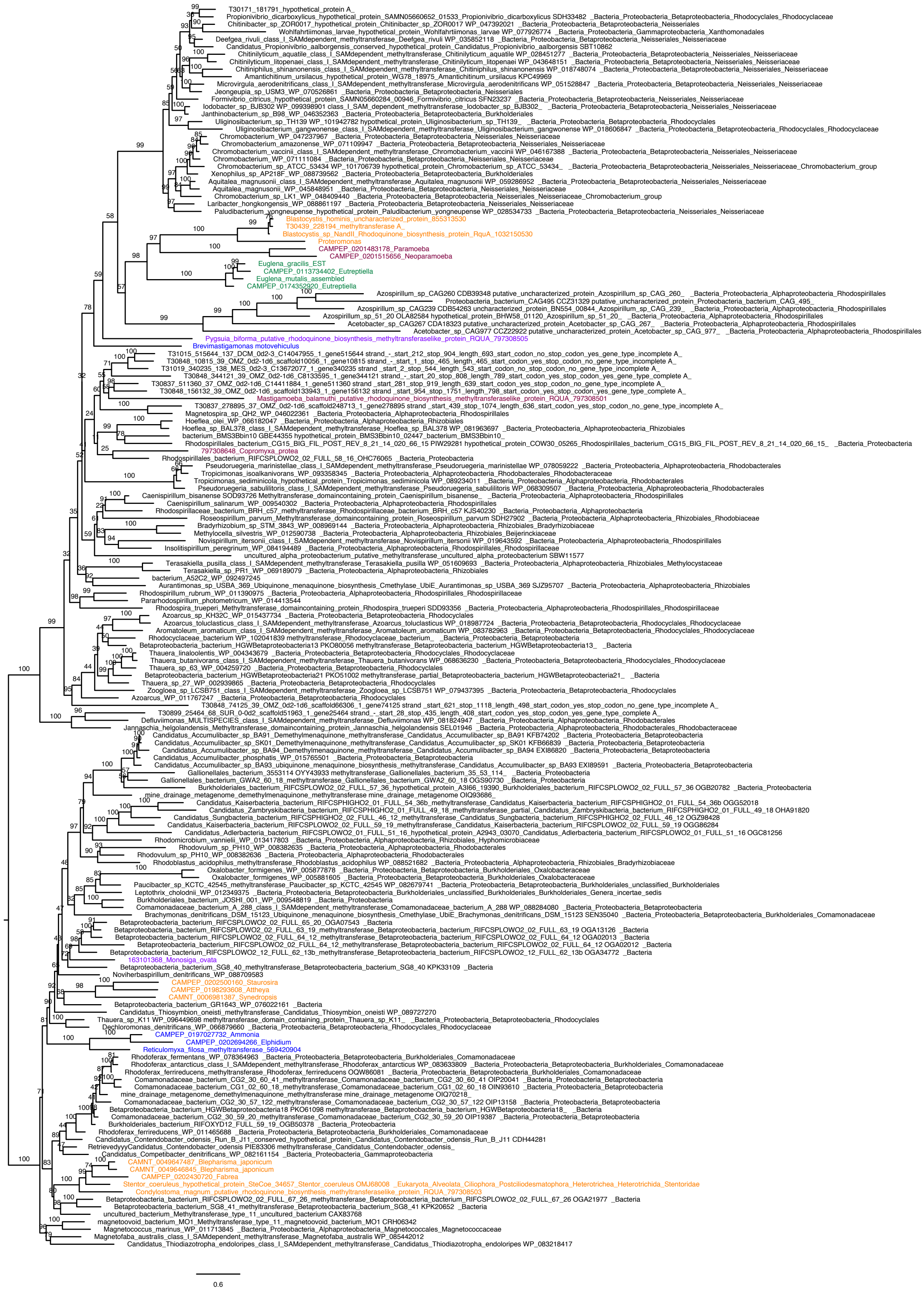

Tree 3 - RQUA Full dataset
Topology Test - Eukaryotic monophyly

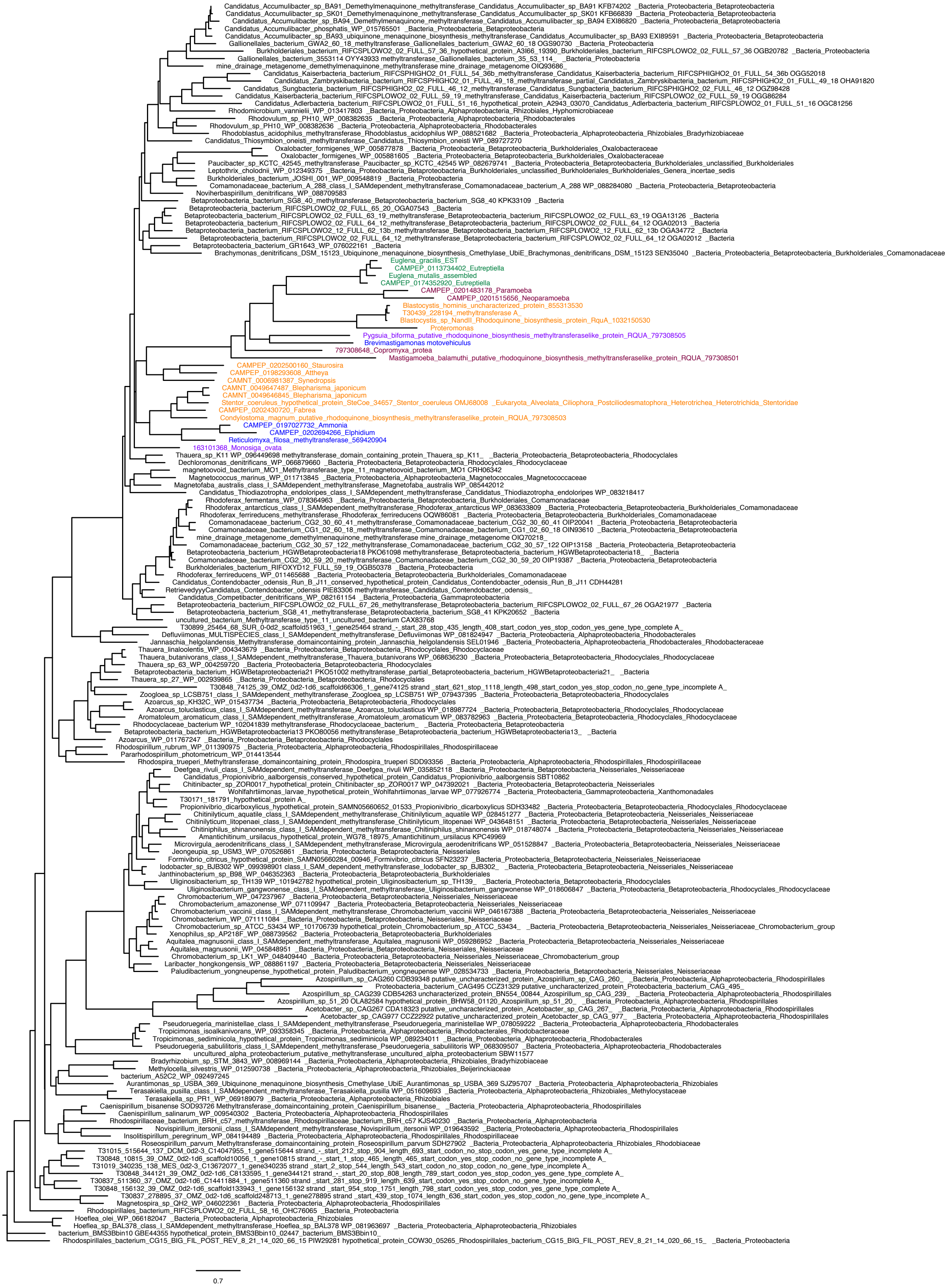

Tree 4 - RQUA Full dataset
Topology Test - Group A1 eukaryotes
Blastocystis, Proteromonas, Neoparamoebids, Euglenids, Pygsuia

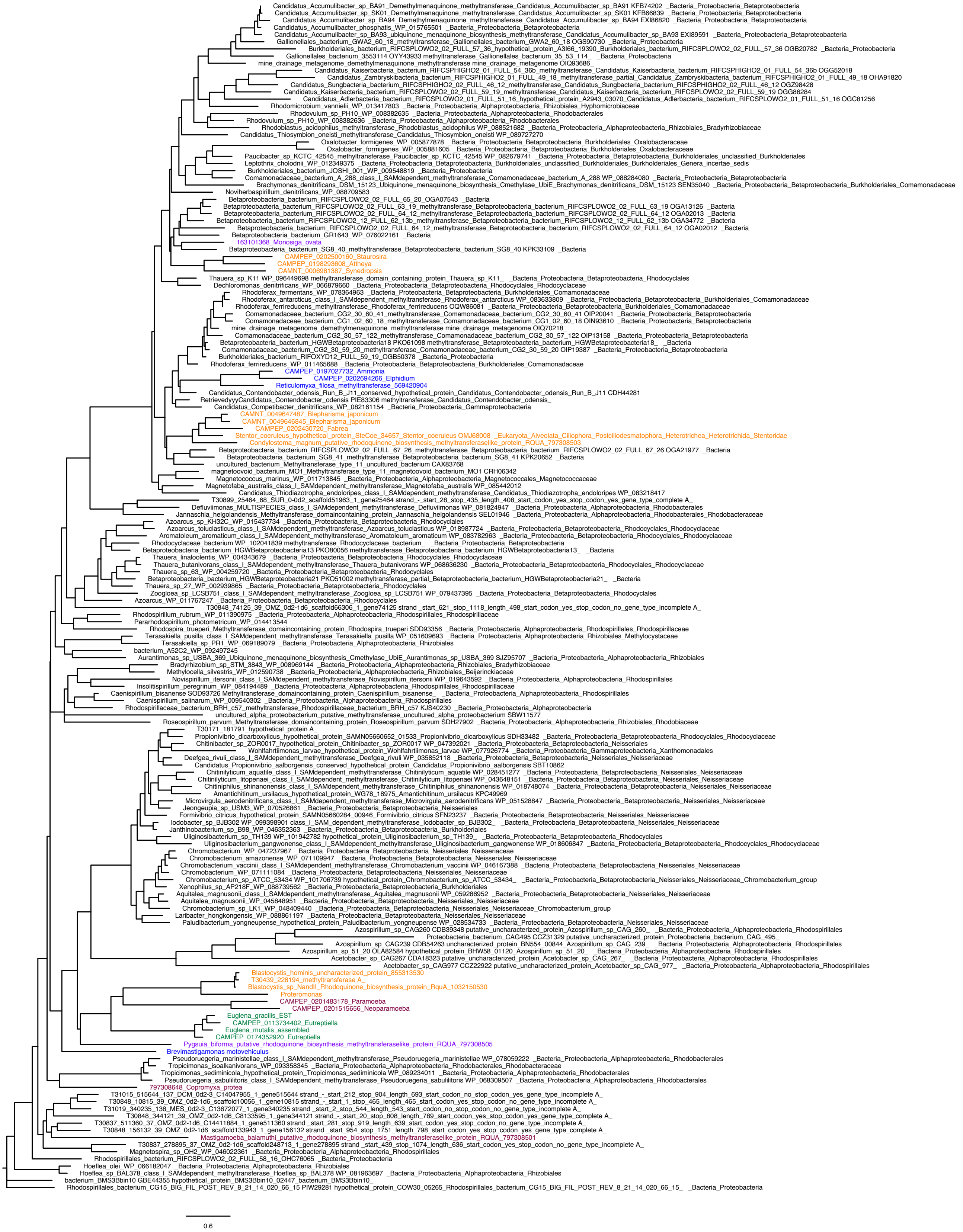

Tree 5 - RQUA Full dataset
Topology Test - Group A1 eukaryotes + Brevimastigamonas

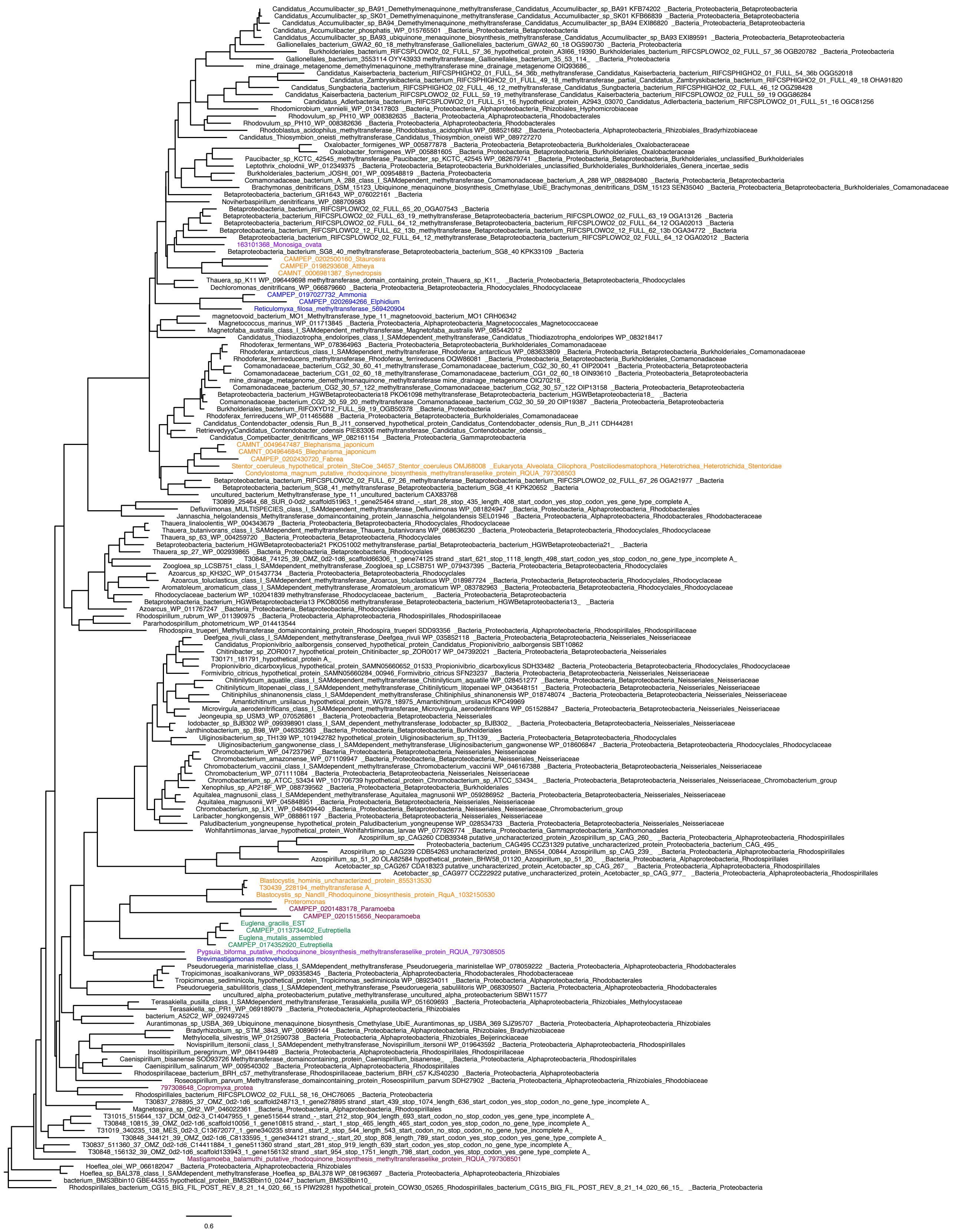

Tree 6 - RQUA Full dataset
Topology Test - Group A1 eukaryotes + *Brevimastigamonas* + *Mastigamoeba*

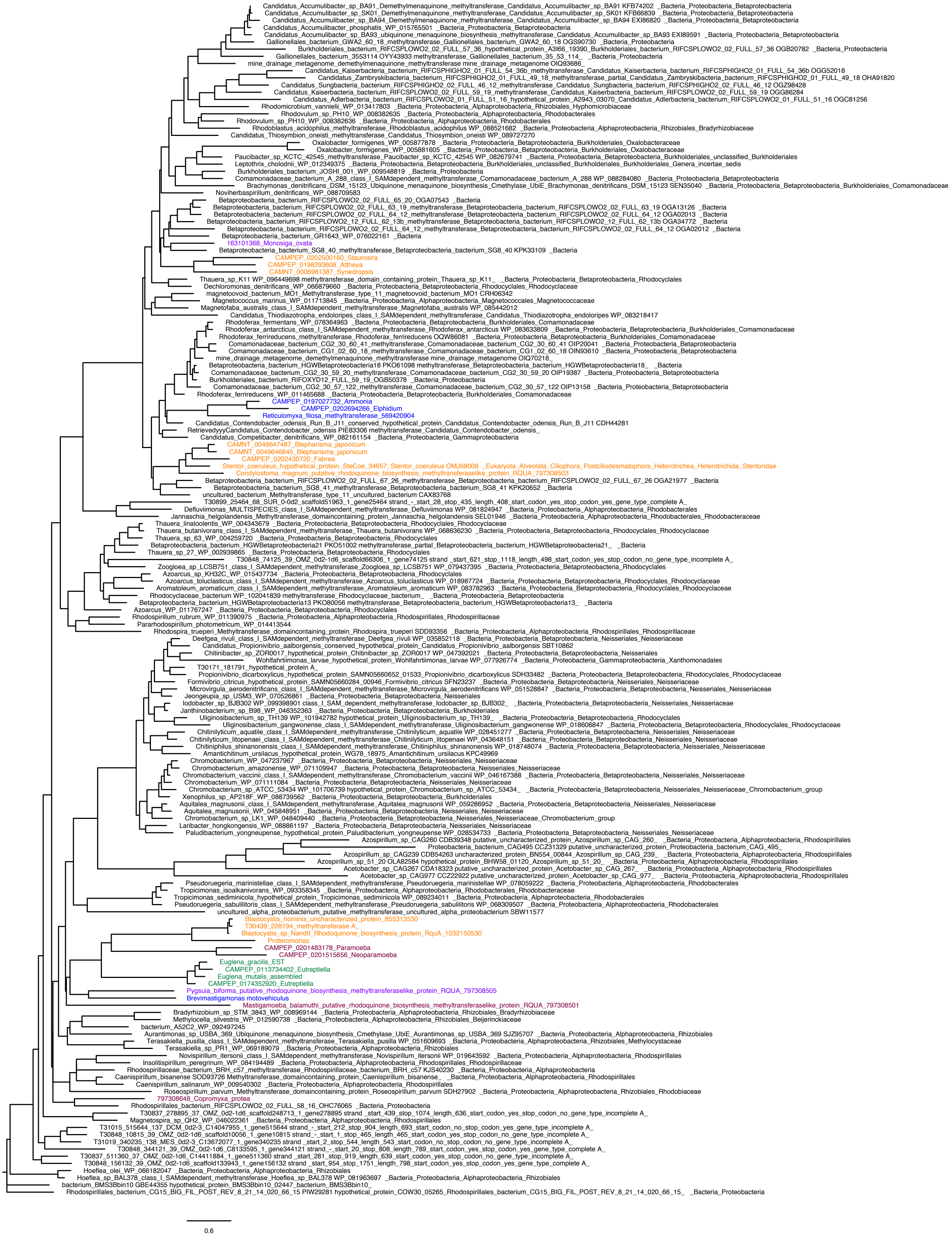

Tree 7 - RQUA Full dataset
Topology Test - Group A eukaryotes

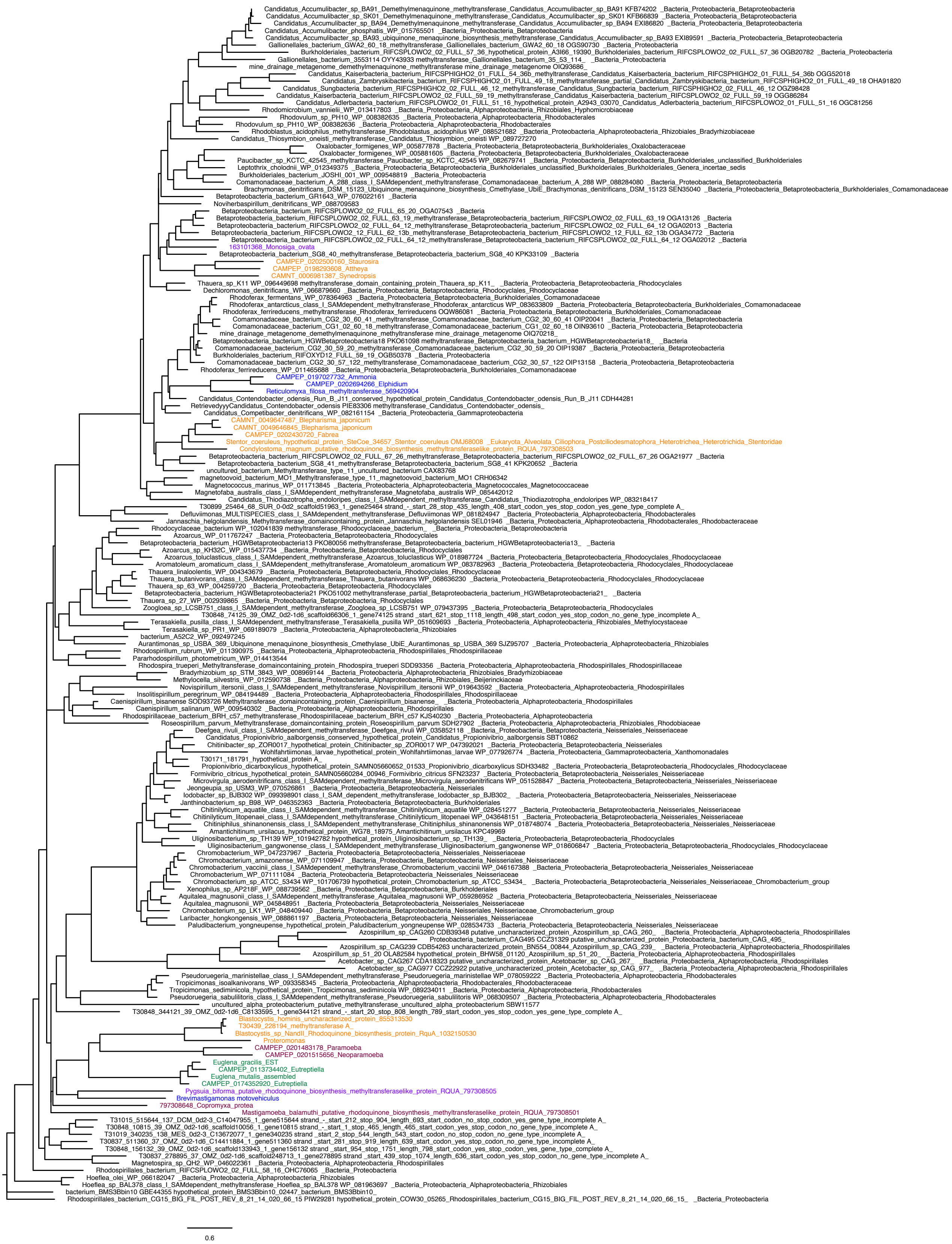

Tree 8 - RQUA Full dataset
Topology Test - Group B eukaryotes

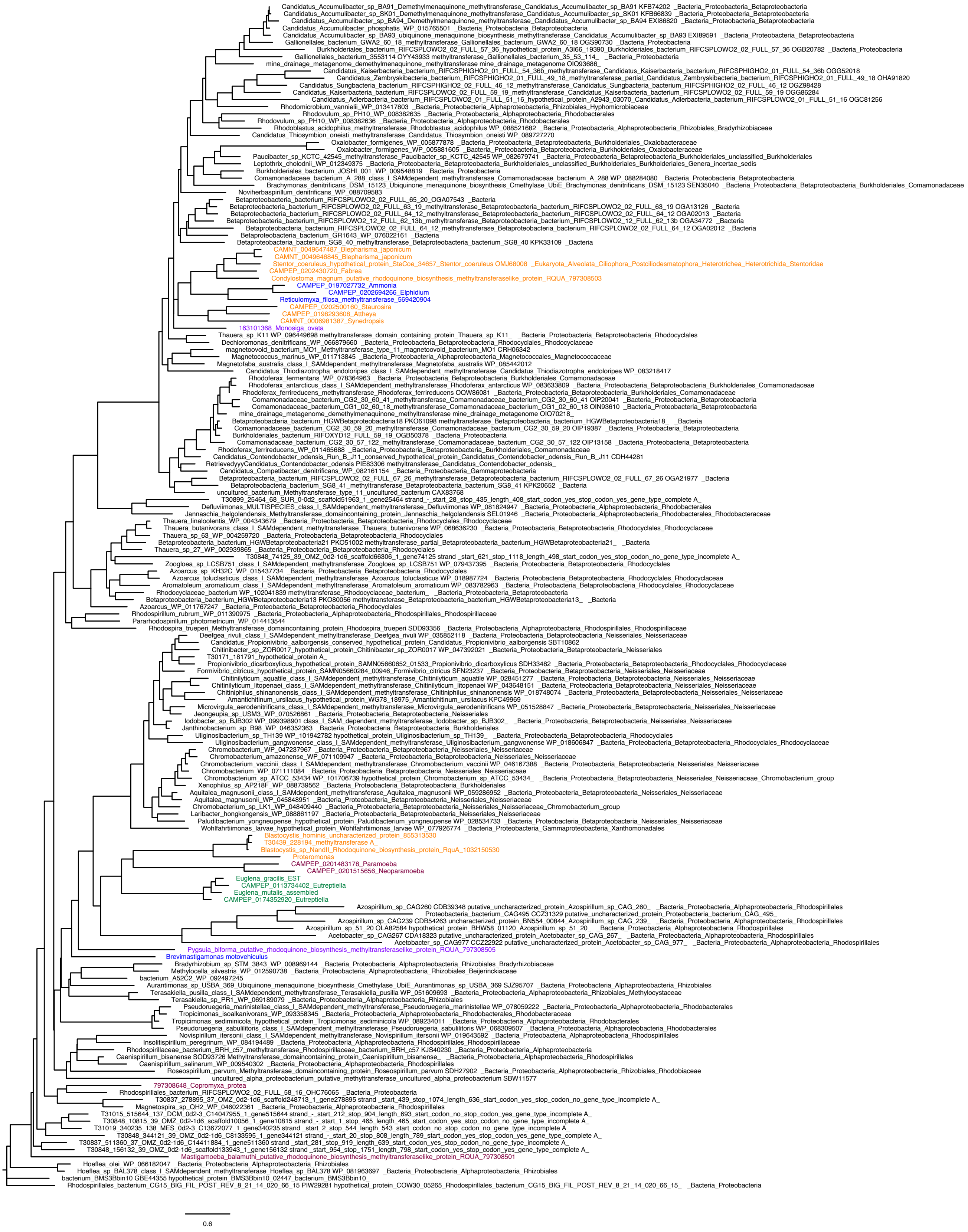

Tree 9 - RQUA Full dataset
Topology Test - Obazoa (Pygsuia + Monosiga)

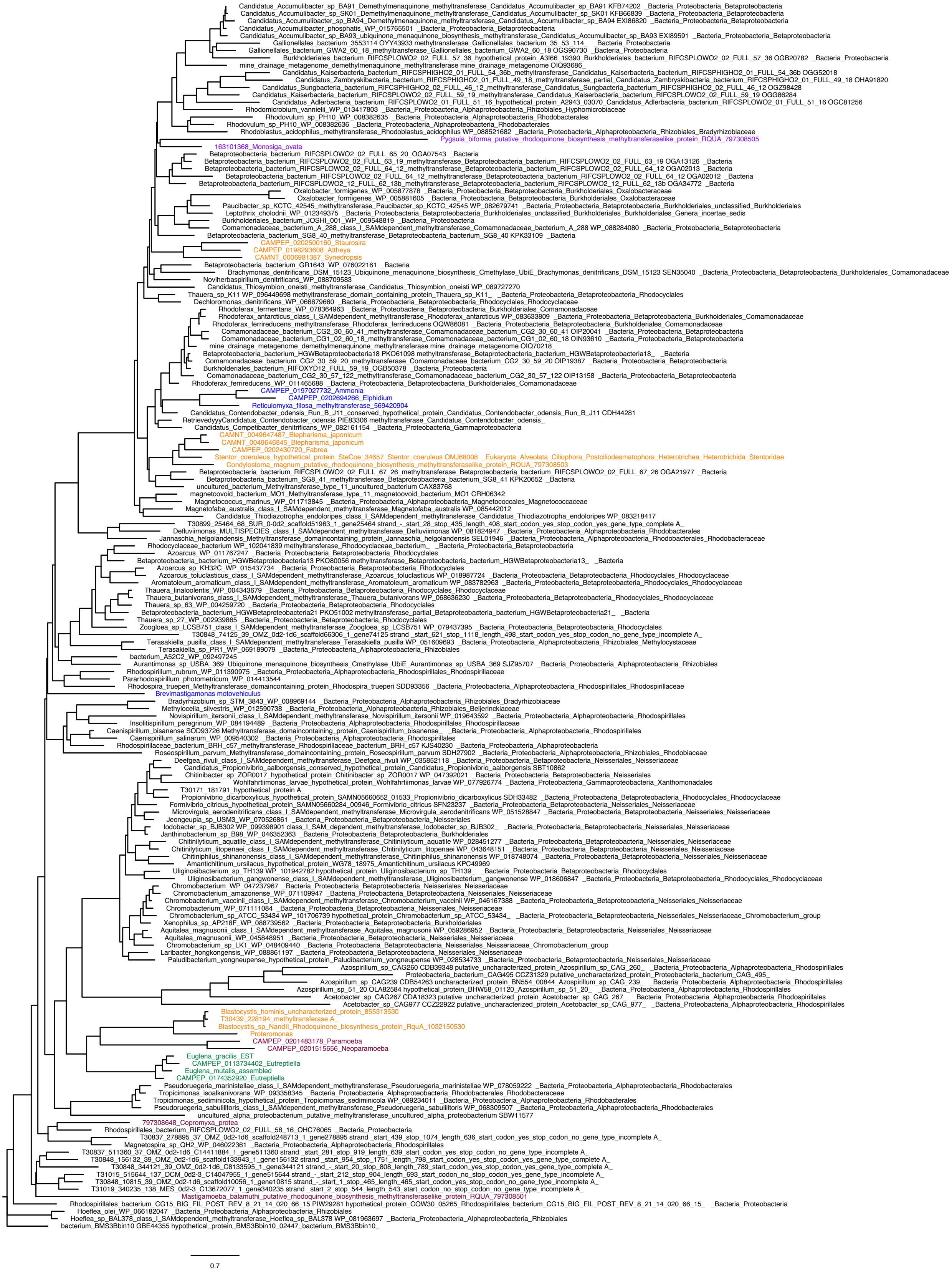

Tree 10 - RQUA Full dataset
Topology Test - Amorphea (Obazoa + Amoebozoa)

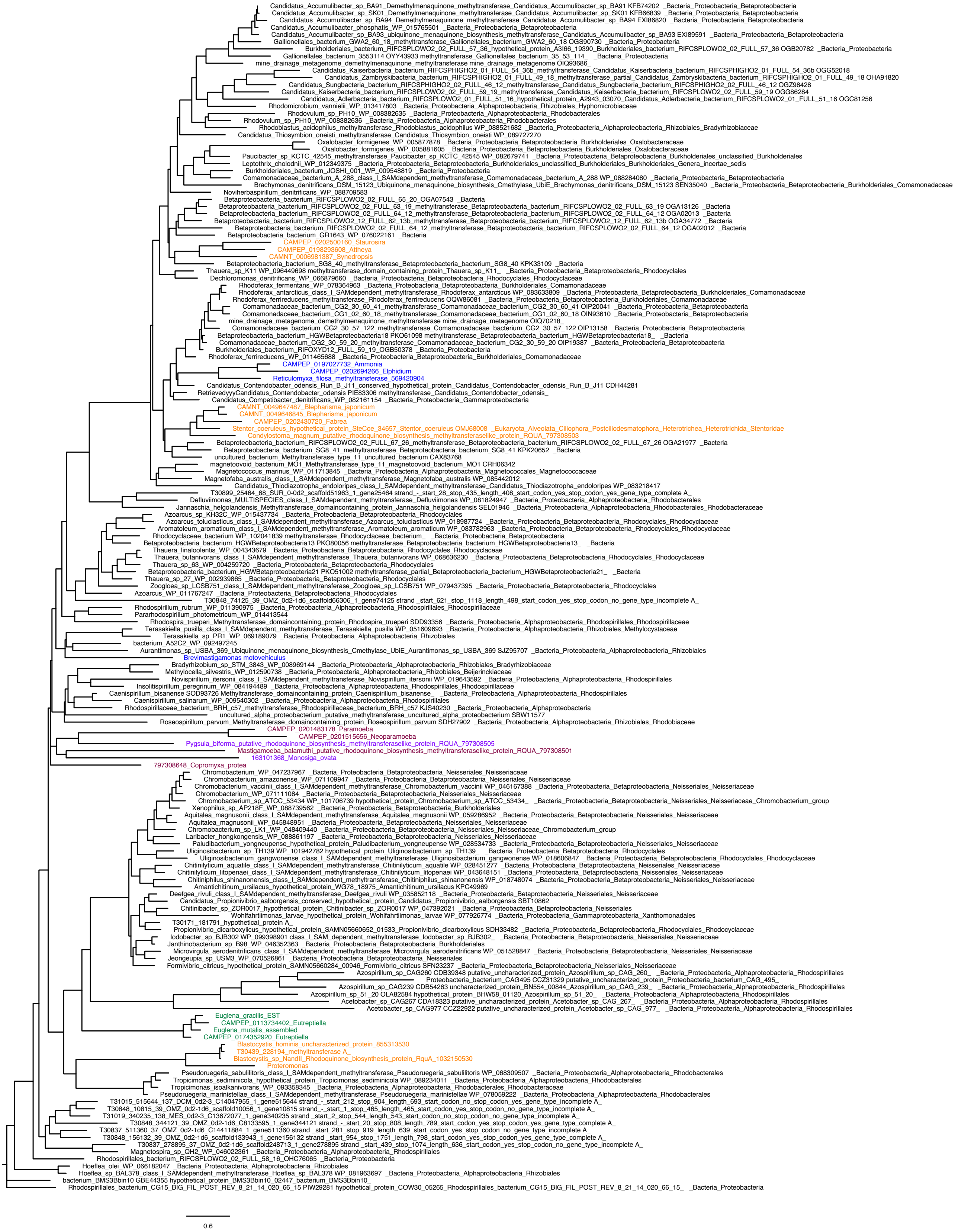

Tree 11 - RQUA Full dataset
Topology Test - Amoebozoa

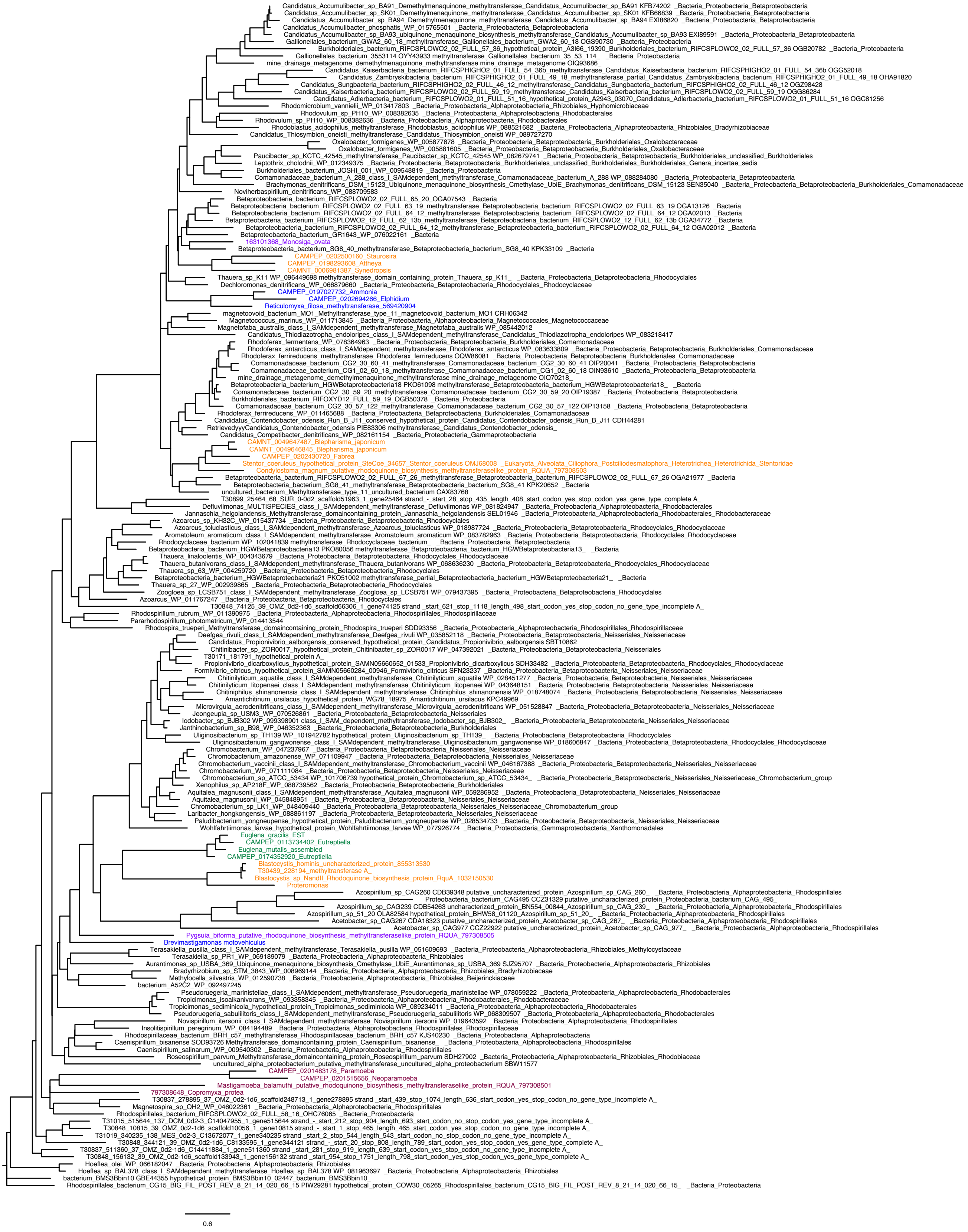

Tree 12 - RQUA Full dataset
Topology Test - Stramenopiles + Alveolates

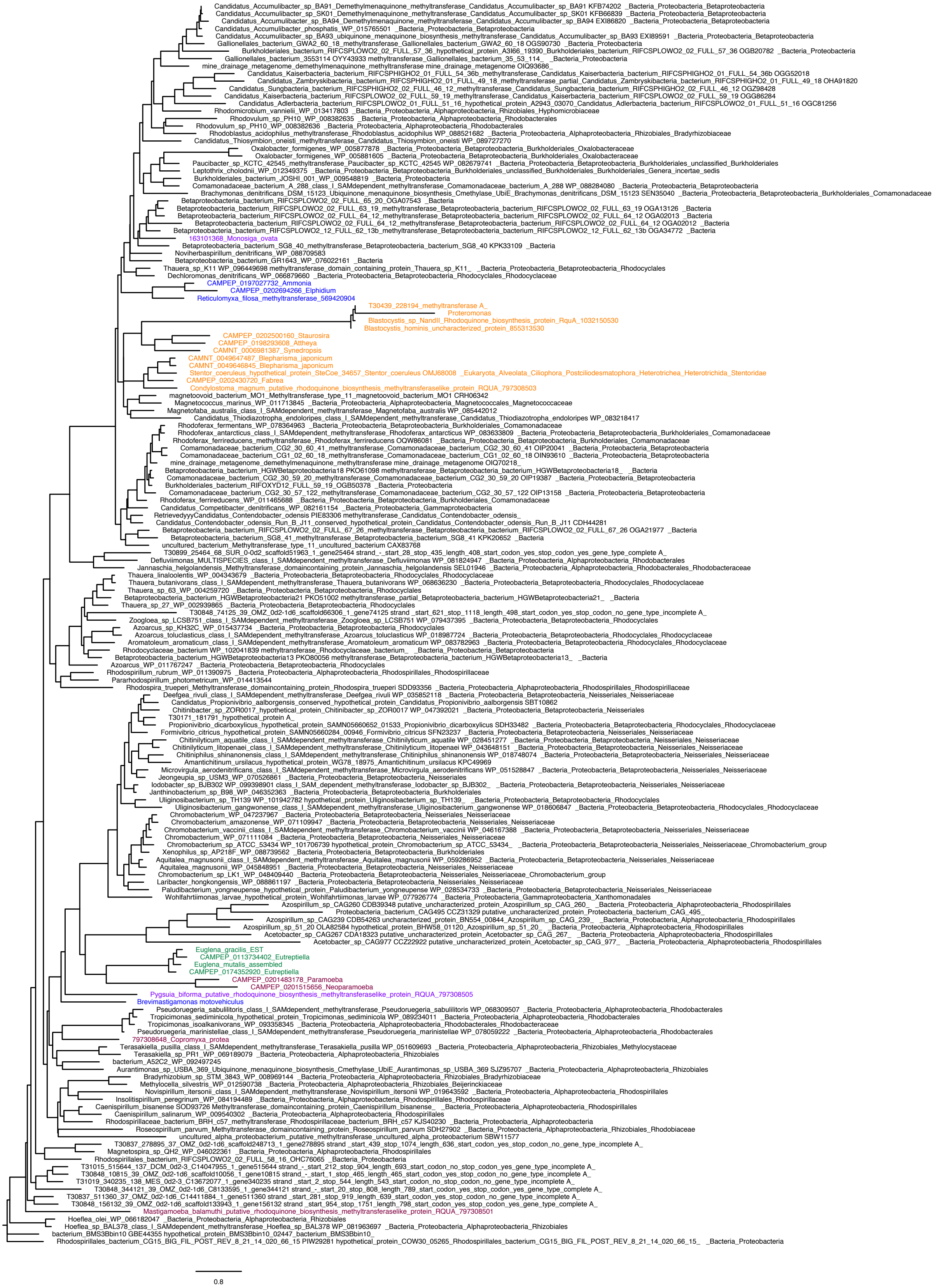

Tree 13 - RQUA Full dataset
Topology Test - Stramenopiles + Alveolates + Rhizaria (SAR)

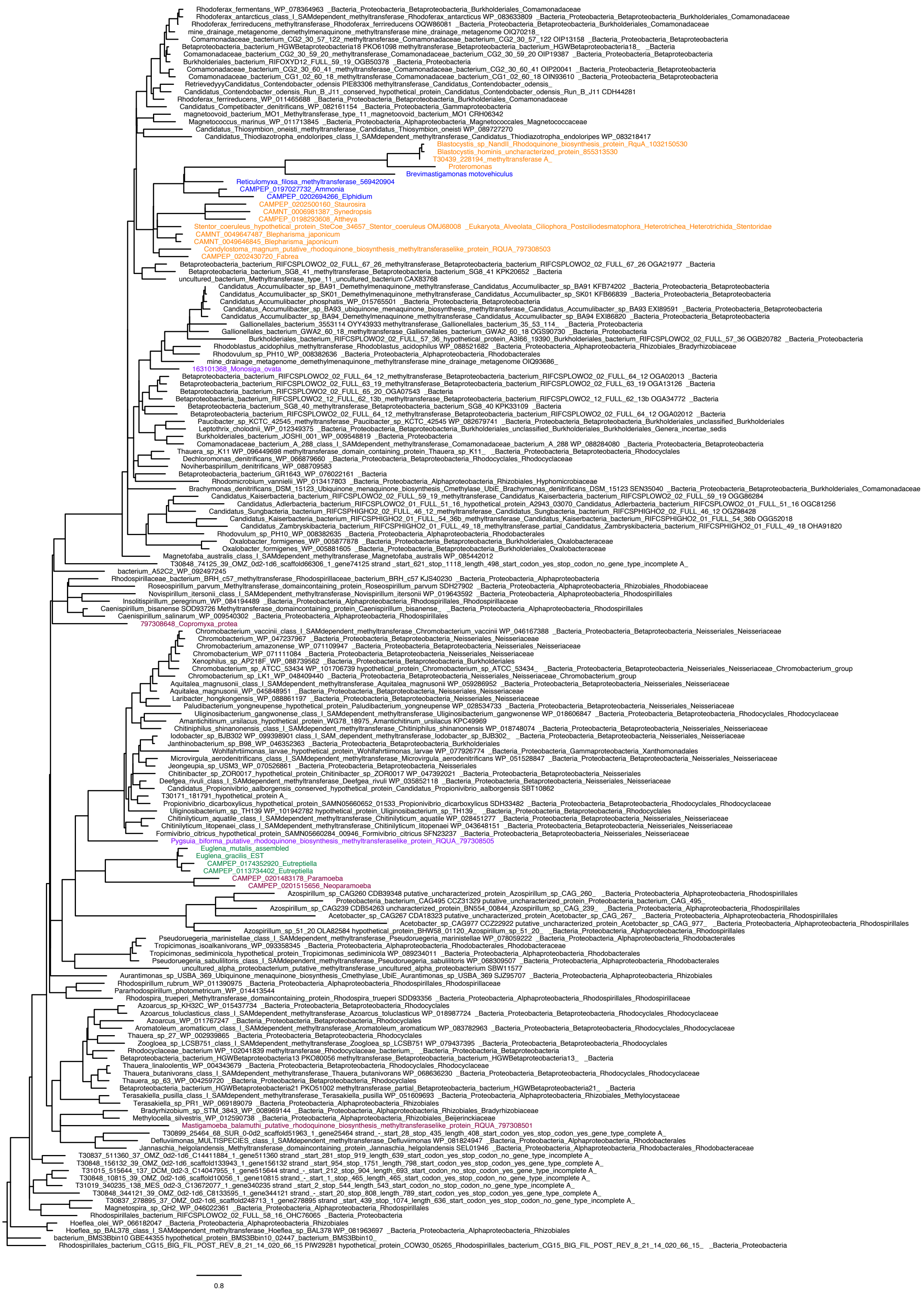

Tree 14 - RQUA Full dataset
Topology Test - Diaphoretickes (SAR + Euglenids)

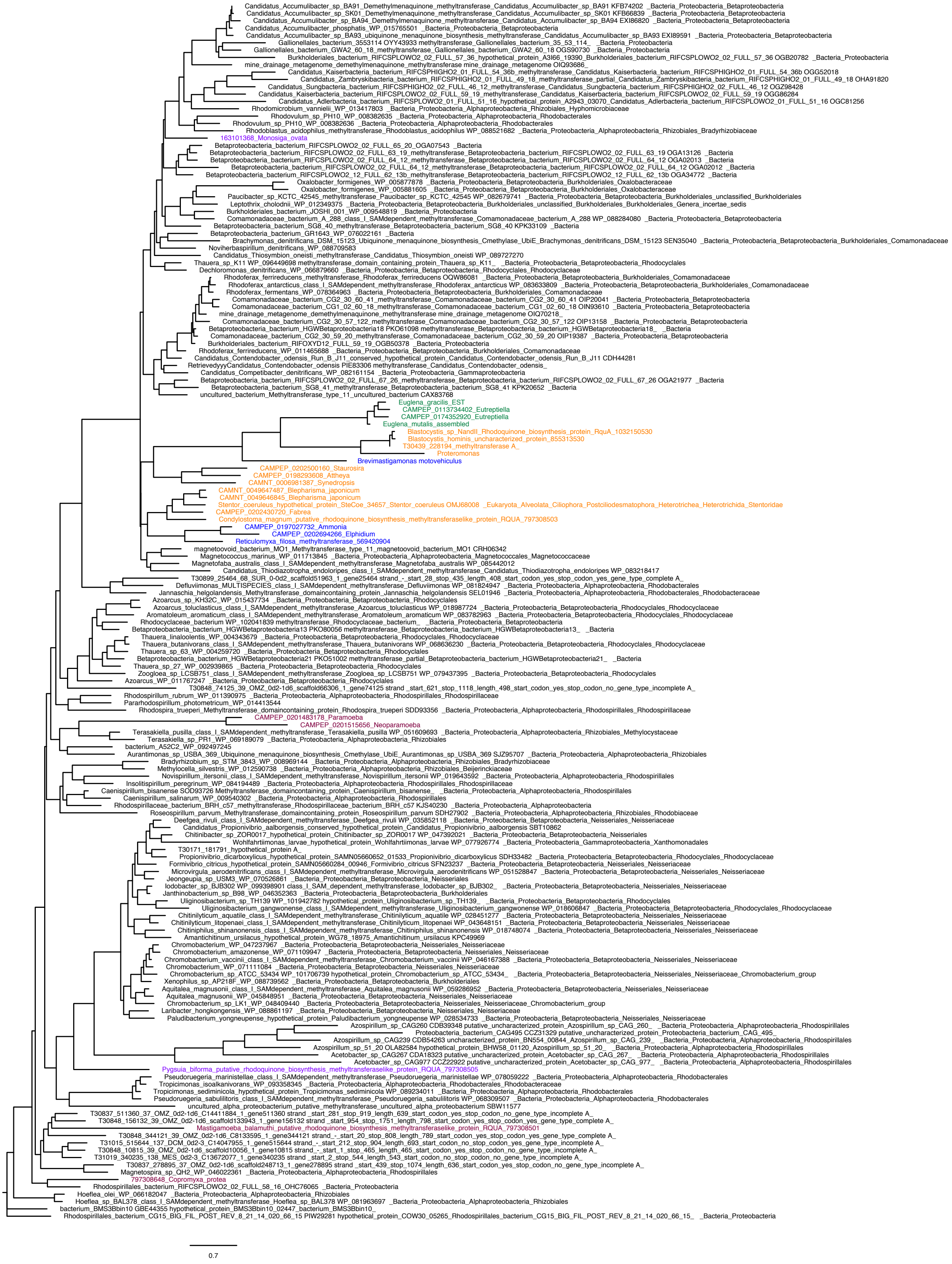

Tree 15 - RQUA Full dataset
Topology Test - Rhizaria

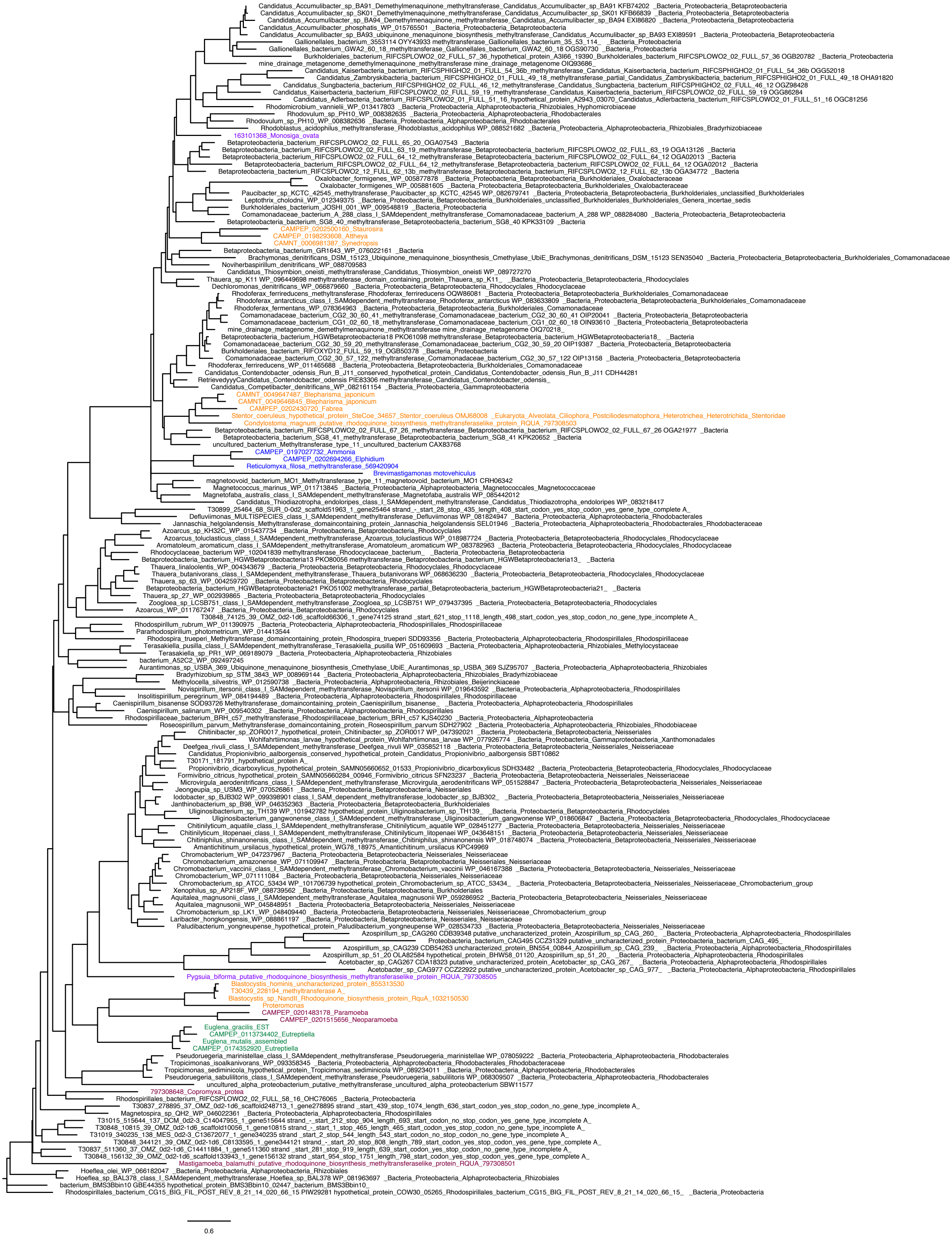

Tree 16 - RQUA Full dataset
Topology Test - Eukaryotes + MAG alphaproteobacteria

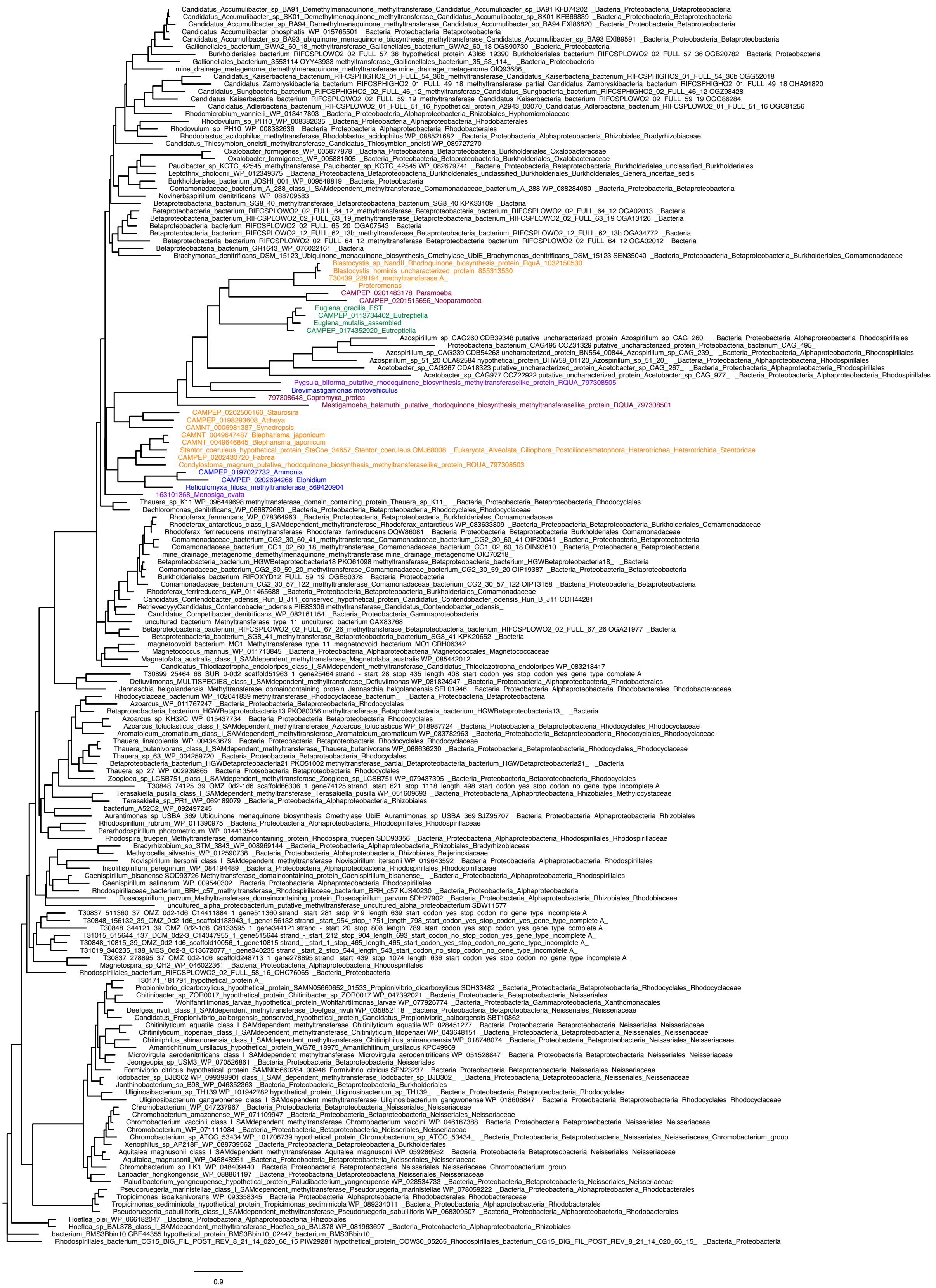

Tree 17 - RQUA Full dataset
Topology Test - Group A eukaryotes + MAG alphaproteobacteria

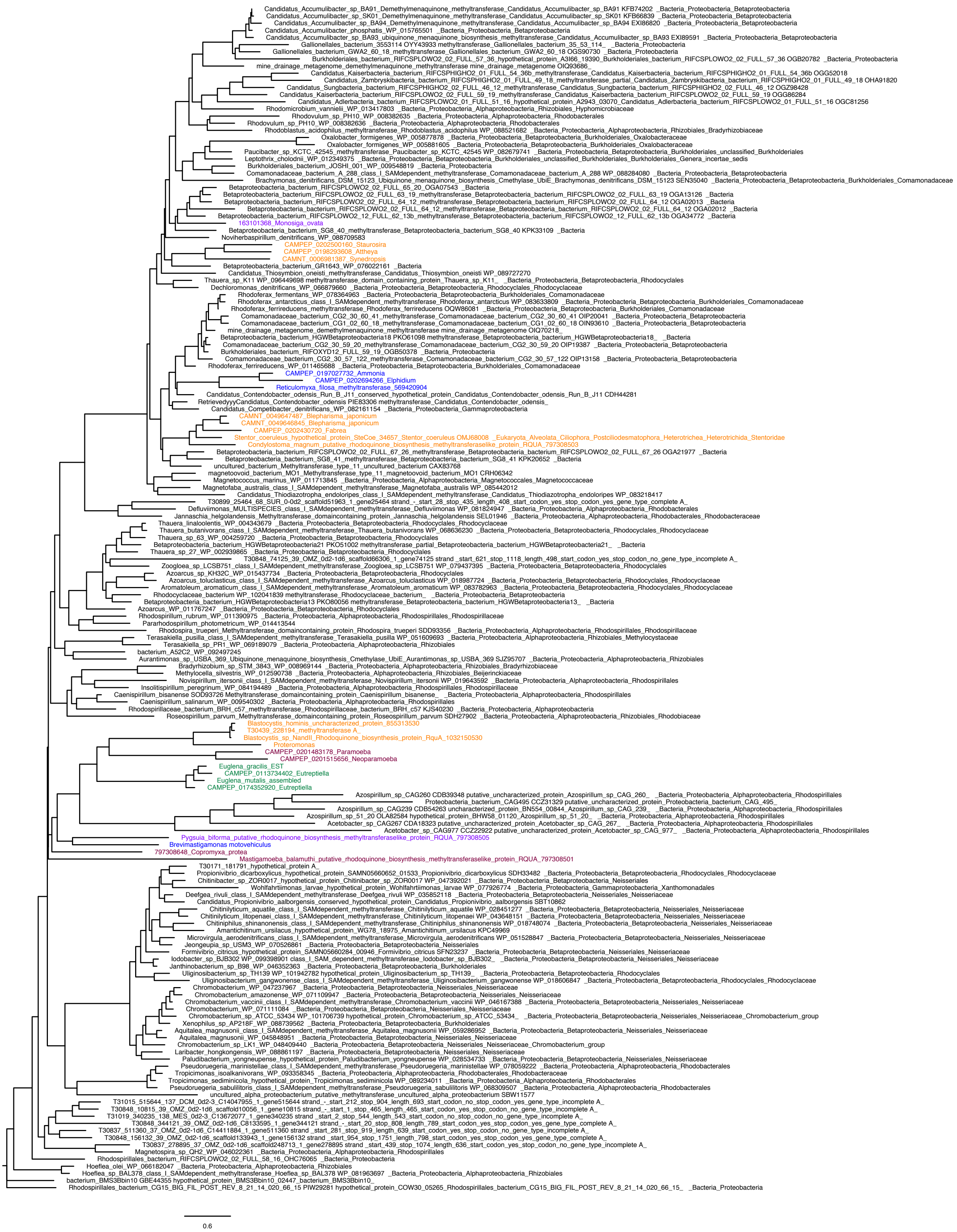

Tree 18 - RQUA Full dataset
Topology Test - alphaproteobacteria

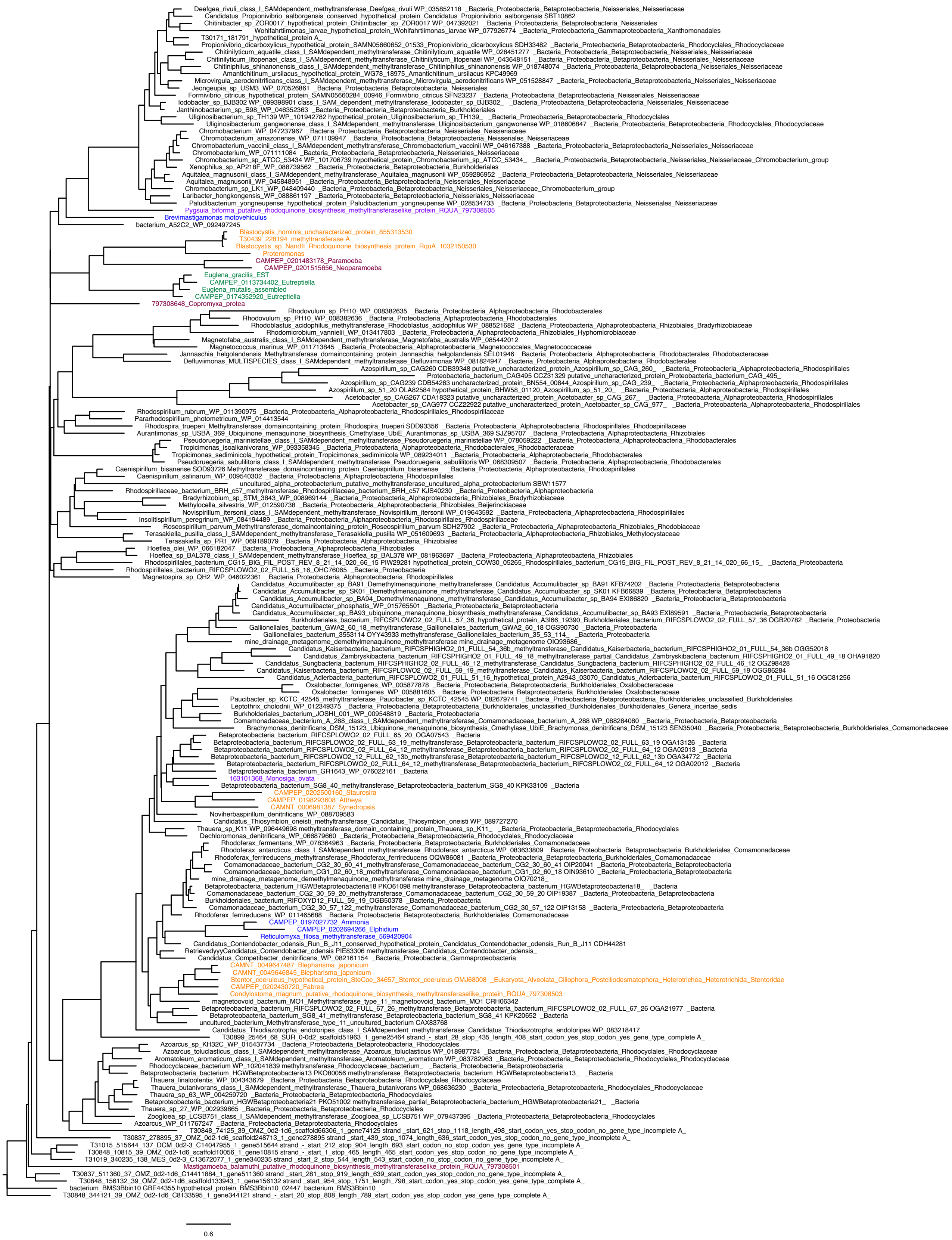

Tree 19 - RQUA Full dataset
Topology Test - alphaproteobacteria + eukaryotes

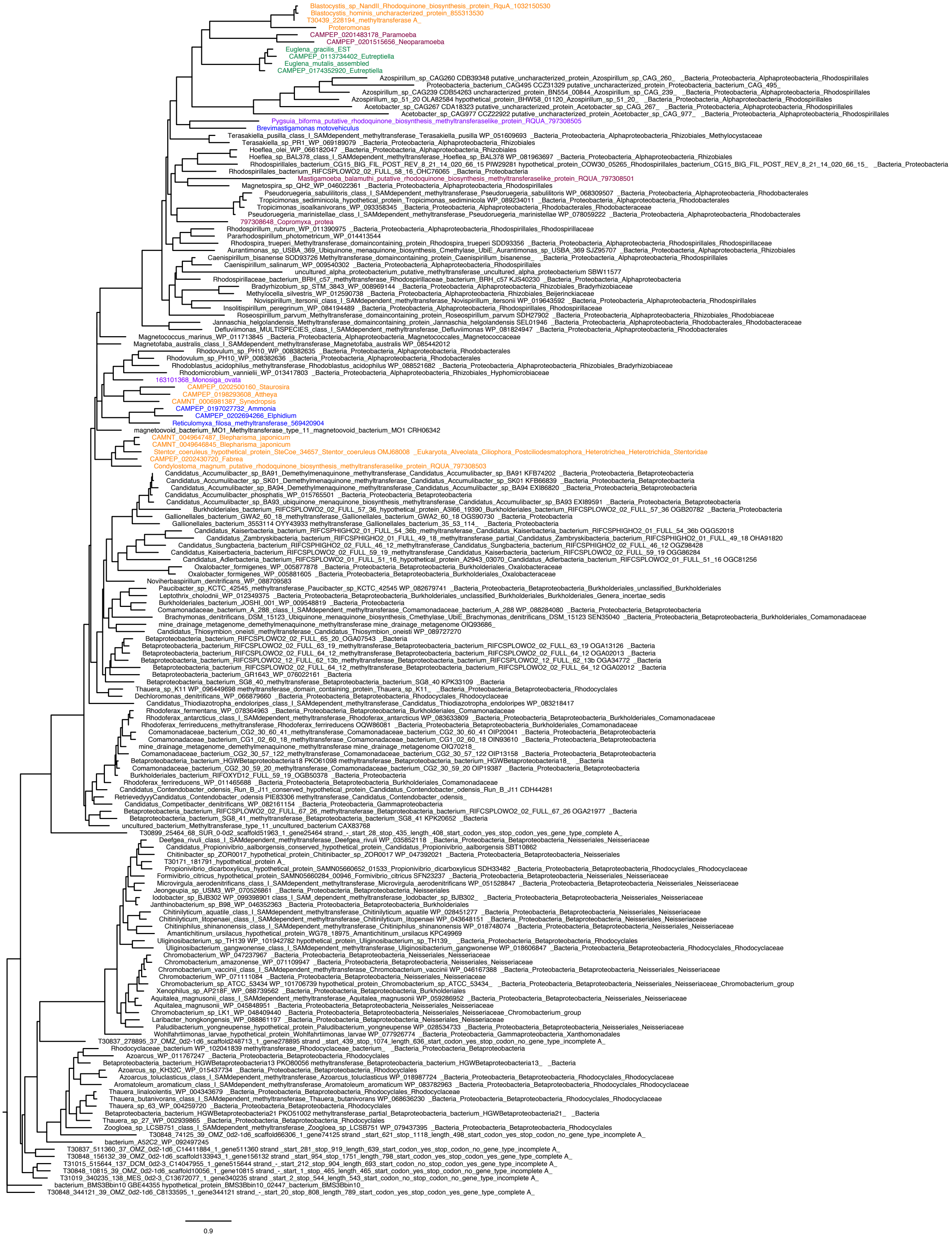

Tree 20 - RQUA Full dataset
Topology Test - Group A eukaryotes + Group A alphaproteobacteria

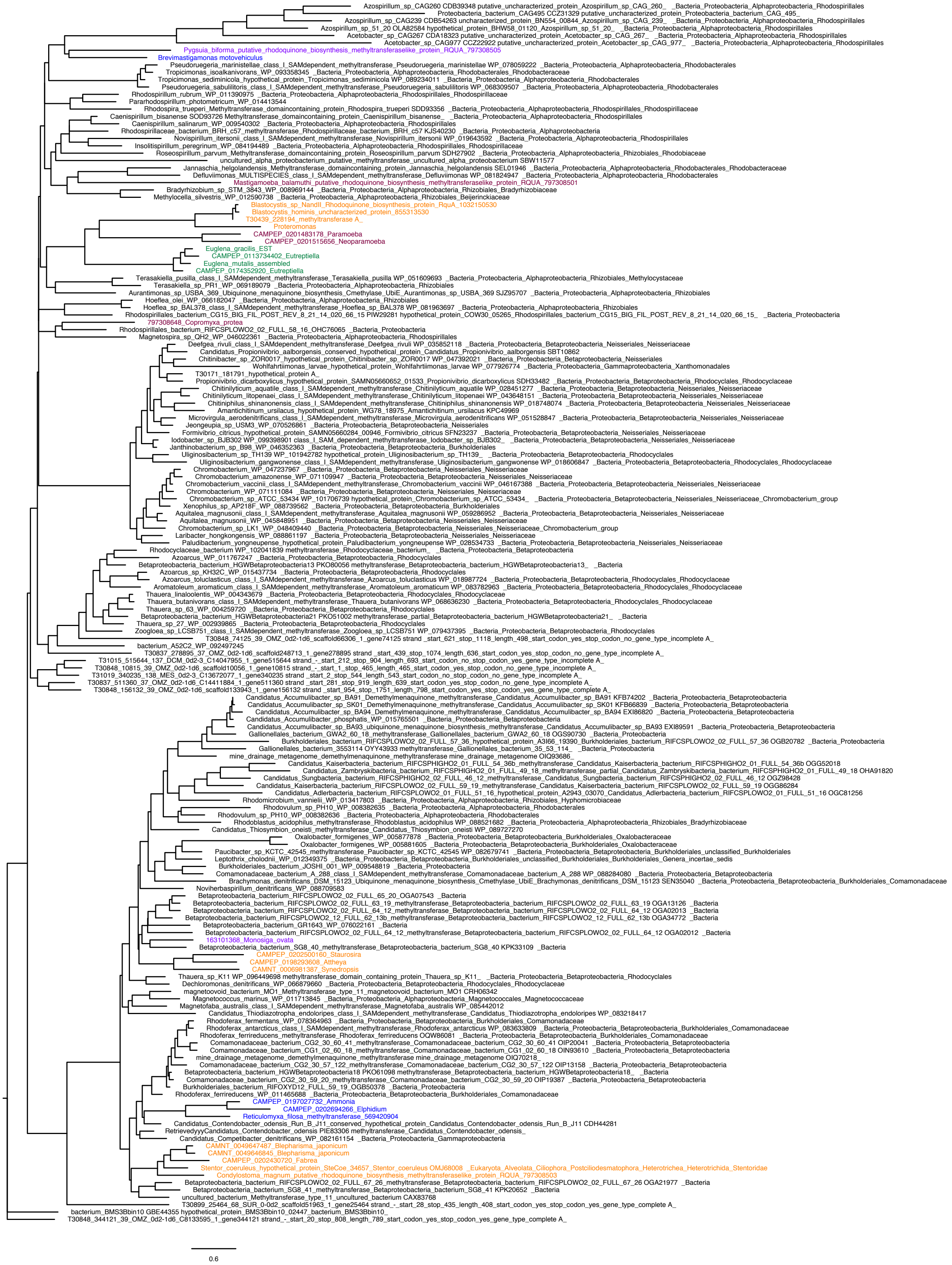

Tree 21 - RQUA Full dataset
Topology Test - Group B eukaryotes + Group B alphaproteobacteria

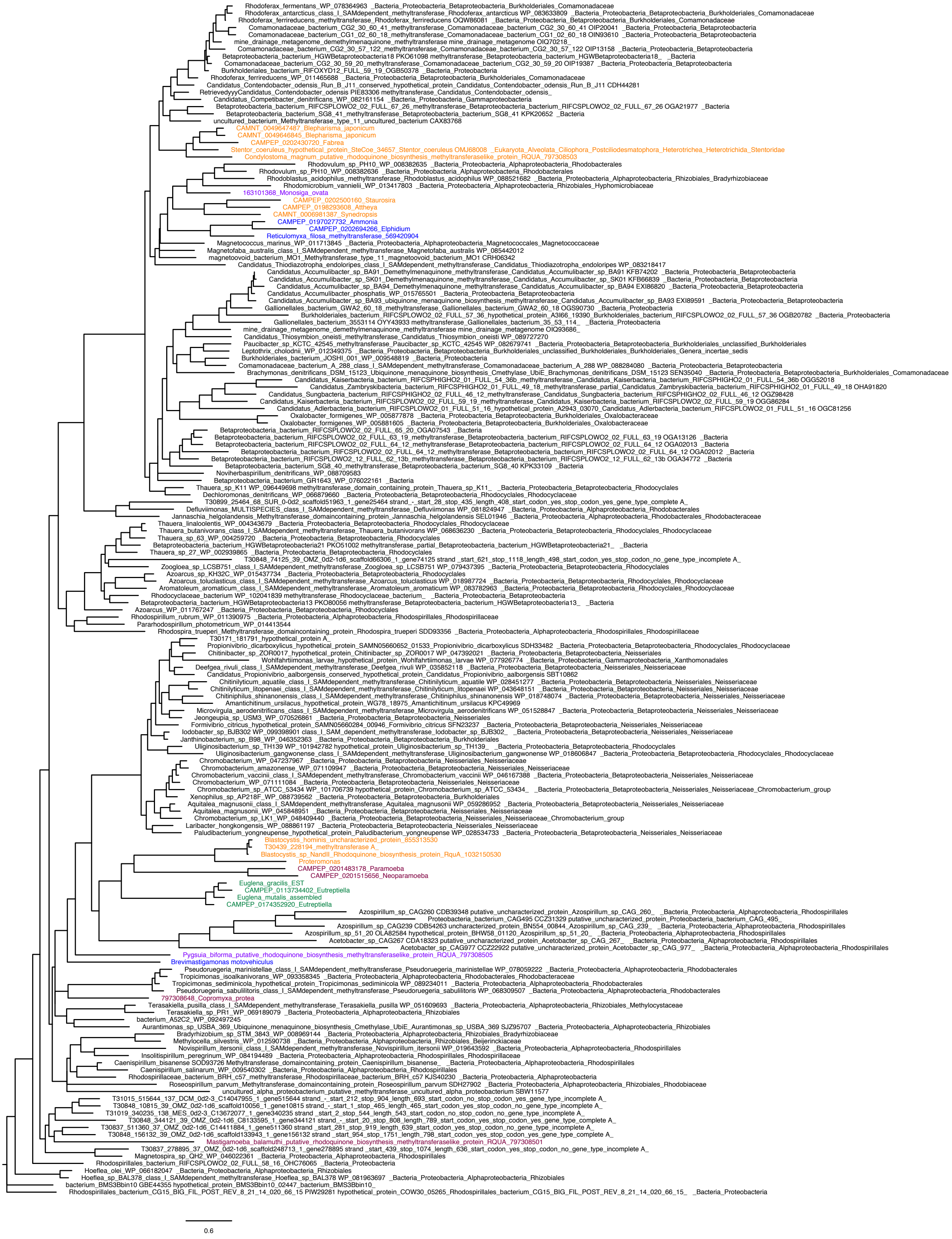

Supplement: Supplementary file 2. [file elife-34292-supp2.pdf]
