# Supplementary material for: Microbial eukaryotes have adapted to hypoxia by horizontal acquisitions of a gene involved in rhodoquinone biosynthesis
Source: eLife. 2018 Apr 26;7:e34292. doi: 10.7554/eLife.34292 (PMC5953543; doi:10.7554/eLife.34292)
Supplement: Supplementary file 3. [file elife-34292-supp3.pdf]

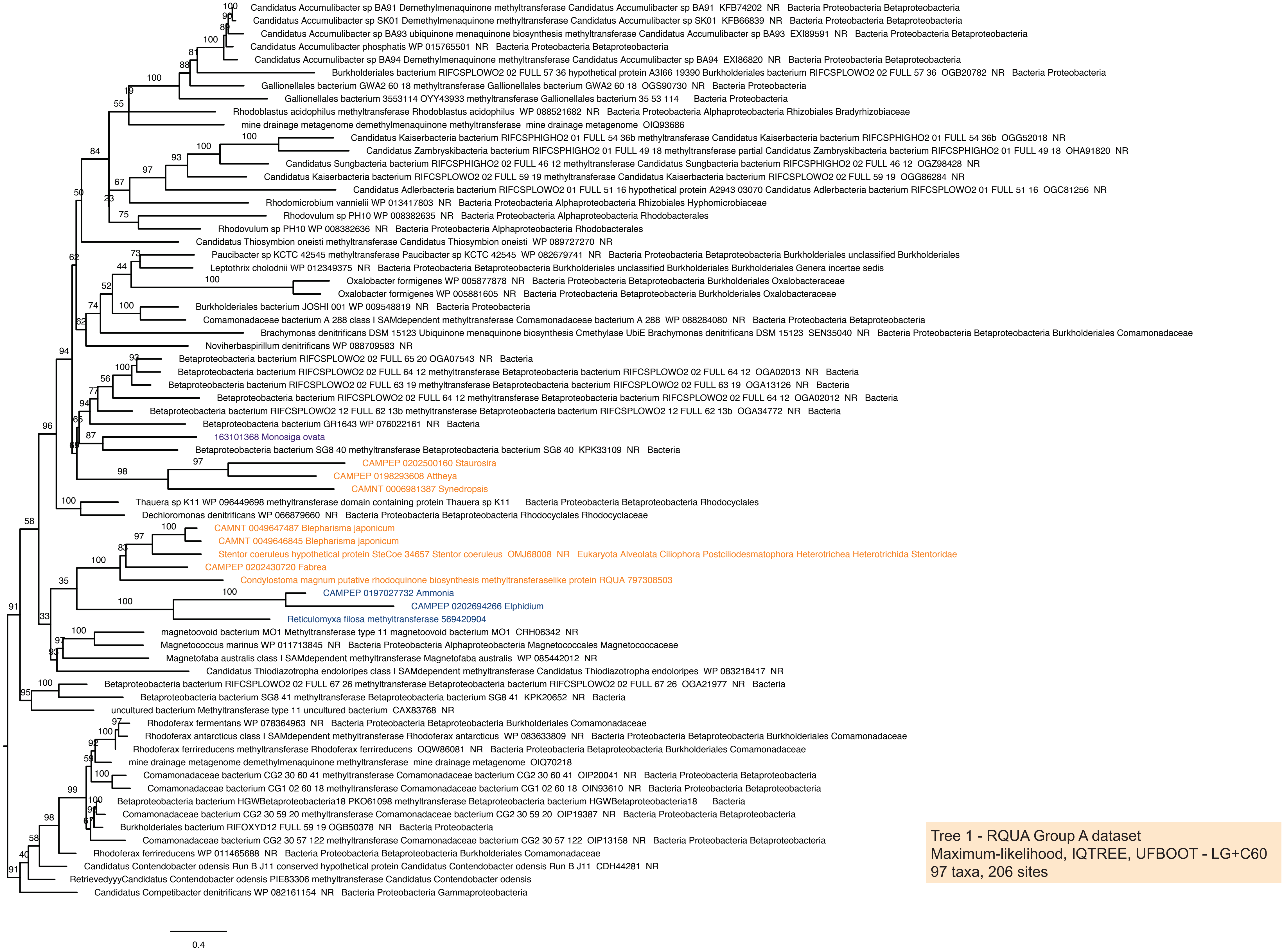
Candidatus Accumulibacter sp BA91 Demethylmenaquinone methyltransferase Candidatus Accumulibacter sp BA91 KFB74202 NR Bacteria Proteobacteria Betaproteobacteria
Candidatus Accumulibacter sp SK01 Demethylmenaquinone methyltransferase Candidatus Accumulibacter sp SK01 KFB66839 NR Bacteria Proteobacteria Betaproteobacteria
Candidatus Accumulibacter sp BA93 ubiquinone menaquinone biosynthesis methyltransferase Candidatus Accumulibacter sp BA93 EXI89591 NR Bacteria Proteobacteria Betaproteobacteria
Candidatus Accumulibacter phosphatis WP 015765501 NR Bacteria Proteobacteria Betaproteobacteria
Candidatus Accumulibacter sp BA94 Demethylmenaquinone methyltransferase Candidatus Accumulibacter sp BA94 EXI86820 NR Bacteria Proteobacteria Betaproteobacteria
Burkholderiales bacterium RIFCSPLOWO2 02 FULL 57 36 hypothetical protein A3I66 19390 Burkholderiales bacterium RIFCSPLOWO2 02 FULL 57 36 OGB20782 NR Bacteria Proteobacteria
Gallionellales bacterium GWA2 60 18 methyltransferase Gallionellales bacterium GWA2 60 18 OGS90730 NR Bacteria Proteobacteria
Gallionellales bacterium 3553114 OYY43933 methyltransferase Gallionellales bacterium 35 53 114 Bacteria Proteobacteria
Rhodoblastus acidophilus methyltransferase Rhodoblastus acidophilus WP 088521682 NR Bacteria Proteobacteria Alphaproteobacteria Rhizobiales Bradyrhizobiaceae
mine drainage metagenome demethylmenaquinone methyltransferase mine drainage metagenome OIQ93686
Candidatus Kaiserbacteria bacterium RIFCSPHIGHO2 01 FULL 54 36b methyltransferase Candidatus Kaiserbacteria bacterium RIFCSPHIGHO2 01 FULL 54 36b OGG52018 NR
Candidatus Zambryskibacteria bacterium RIFCSPHIGHO2 01 FULL 49 18 methyltransferase partial Candidatus Zambryskibacteria bacterium RIFCSPHIGHO2 01 FULL 49 18 OHA91820 NR
Candidatus Sungbacteria bacterium RIFCSPHIGHO2 02 FULL 46 12 methyltransferase Candidatus Sungbacteria bacterium RIFCSPHIGHO2 02 FULL 46 12 OGZ98428 NR
Candidatus Kaiserbacteria bacterium RIFCSPLOWO2 02 FULL 59 19 methyltransferase Candidatus Kaiserbacteria bacterium RIFCSPLOWO2 02 FULL 59 19 OGG86284 NR
Candidatus Adlerbacteria bacterium RIFCSPLOWO2 01 FULL 51 16 hypothetical protein A2943 03070 Candidatus Adlerbacteria bacterium RIFCSPLOWO2 01 FULL 51 16 OGC81256 NR
Rhodomicrobium vannielii WP 013417803 NR Bacteria Proteobacteria Alphaproteobacteria Rhizobiales Hyphomicrobiaceae
Rhodovulum sp PH10 WP 008382635 NR Bacteria Proteobacteria Alphaproteobacteria Rhodobacterales
Rhodovulum sp PH10 WP 008382636 NR Bacteria Proteobacteria Alphaproteobacteria Rhodobacterales
Candidatus Thiosymbion oneisti methyltransferase Candidatus Thiosymbion oneisti WP 089727270 NR
Paucibacter sp KCTC 42545 methyltransferase Paucibacter sp KCTC 42545 WP 082679741 NR Bacteria Proteobacteria Betaproteobacteria Burkholderiales unclassified Burkholderiales
Leptothrix cholodnii WP 012349375 NR Bacteria Proteobacteria Betaproteobacteria Burkholderiales unclassified Burkholderiales Burkholderiales Genera incertae sedis
Oxalobacter formigenes WP 005877878 NR Bacteria Proteobacteria Betaproteobacteria Burkholderiales Oxalobacteraceae
Oxalobacter formigenes WP 005881605 NR Bacteria Proteobacteria Betaproteobacteria Burkholderiales Oxalobacteraceae
Burkholderiales bacterium JOSHI 001 WP 009548819 NR Bacteria Proteobacteria
Comamonadaceae bacterium A 288 class I SAMdependent methyltransferase Comamonadaceae bacterium A 288 WP 088284080 NR Bacteria Proteobacteria Betaproteobacteria
Brachymonas denitrificans DSM 15123 Ubiquinone menaquinone biosynthesis Cmethylase UbiE Brachymonas denitrificans DSM 15123 SEN35040 NR Bacteria Proteobacteria Betaproteobacteria Burkholderiales Comamonadaceae
Noviherbaspirillum denitrificans WP 088709583 NR
Betaproteobacteria bacterium RIFCSPLOWO2 02 FULL 65 20 OGA07543 NR Bacteria
Betaproteobacteria bacterium RIFCSPLOWO2 02 FULL 64 12 methyltransferase Betaproteobacteria bacterium RIFCSPLOWO2 02 FULL 64 12 OGA02013 NR Bacteria
Betaproteobacteria bacterium RIFCSPLOWO2 02 FULL 63 19 methyltransferase Betaproteobacteria bacterium RIFCSPLOWO2 02 FULL 63 19 OGA13126 NR Bacteria
Betaproteobacteria bacterium RIFCSPLOWO2 02 FULL 64 12 methyltransferase Betaproteobacteria bacterium RIFCSPLOWO2 02 FULL 64 12 OGA02012 NR Bacteria
Betaproteobacteria bacterium RIFCSPLOWO2 12 FULL 62 13b methyltransferase Betaproteobacteria bacterium RIFCSPLOWO2 12 FULL 62 13b OGA34772 NR Bacteria
Betaproteobacteria bacterium GR1643 WP 076022161 NR Bacteria
163101368 Monosiga ovata
Betaproteobacteria bacterium SG8 40 methyltransferase Betaproteobacteria bacterium SG8 40 KPK33109 NR Bacteria
CAMPEP 0202500160 Staurosira
CAMPEP 0198293608 Attheya
CAMNT 0006981387 Synedropsis
Thauera sp K11 WP 096449698 methyltransferase domain containing protein Thauera sp K11 Bacteria Proteobacteria Betaproteobacteria Rhodocyclales
Dechloromonas denitrificans WP 066879660 NR Bacteria Proteobacteria Betaproteobacteria Rhodocyclales Rhodocyclaceae
CAMNT 0049647487 Blepharisma japonicum
CAMNT 0049646845 Blepharisma japonicum
Stentor coeruleus hypothetical protein SteCoe 34657 Stentor coeruleus OMJ68008 NR Eukaryota Alveolata Ciliophora Postciliodesmatophora Heterotrichea Heterotrichida Stentoridae
CAMPEP 0202430720 Fabrea
Condylostoma magnum putative rhodoquinone biosynthesis methyltransferaselike protein RQUA 797308503
CAMPEP 0197027732 Ammonia
CAMPEP 0202694266 Elphidium
Reticulomyxa filosa methyltransferase 569420904
magnetoovoid bacterium MO1 Methyltransferase type 11 magnetoovoid bacterium MO1 CRH06342 NR
Magnetococcus marinus WP 011713845 NR Bacteria Proteobacteria Alphaproteobacteria Magnetococcales Magnetococcaceae
Magnetofaba australis class I SAMdependent methyltransferase Magnetofaba australis WP 085442012 NR
Candidatus Thiodiazotropha endoloripes class I SAMdependent methyltransferase Candidatus Thiodiazotropha endoloripes WP 083218417 NR
Betaproteobacteria bacterium RIFCSPLOWO2 02 FULL 67 26 methyltransferase Betaproteobacteria bacterium RIFCSPLOWO2 02 FULL 67 26 OGA21977 NR Bacteria
Betaproteobacteria bacterium SG8 41 methyltransferase Betaproteobacteria bacterium SG8 41 KPK20652 NR Bacteria
uncultured bacterium Methyltransferase type 11 uncultured bacterium CAX83768 NR
Rhodoferax fermentans WP 078364963 NR Bacteria Proteobacteria Betaproteobacteria Burkholderiales Comamonadaceae
Rhodoferax antarcticus class I SAMdependent methyltransferase Rhodoferax antarcticus WP 083633809 NR Bacteria Proteobacteria Betaproteobacteria Burkholderiales Comamonadaceae
Rhodoferax ferrireducens methyltransferase Rhodoferax ferrireducens OQW86081 NR Bacteria Proteobacteria Betaproteobacteria Burkholderiales Comamonadaceae
mine drainage metagenome demethylmenaquinone methyltransferase mine drainage metagenome OIQ70218
Comamonadaceae bacterium CG2 30 60 41 methyltransferase Comamonadaceae bacterium CG2 30 60 41 OIP20041 NR Bacteria Proteobacteria Betaproteobacteria
Comamonadaceae bacterium CG1 02 60 18 methyltransferase Comamonadaceae bacterium CG1 02 60 18 OIN93610 NR Bacteria Proteobacteria Betaproteobacteria
Betaproteobacteria bacterium HGWBetaproteobacteria18 PKO61098 methyltransferase Betaproteobacteria bacterium HGWBetaproteobacteria18 Bacteria
Comamonadaceae bacterium CG2 30 59 20 methyltransferase Comamonadaceae bacterium CG2 30 59 20 OIP19387 NR Bacteria Proteobacteria Betaproteobacteria
Burkholderiales bacterium RIFOXYD12 FULL 59 19 OGB50378 NR Bacteria Proteobacteria
Comamonadaceae bacterium CG2 30 57 122 methyltransferase Comamonadaceae bacterium CG2 30 57 122 OIP13158 NR Bacteria Proteobacteria Betaproteobacteria
Rhodoferax ferrireducens WP 011465688 NR Bacteria Proteobacteria Betaproteobacteria Burkholderiales Comamonadaceae
Candidatus Contendobacter odensis Run B J11 conserved hypothetical protein Candidatus Contendobacter odensis Run B J11 CDH44281 NR
RetrievedyyyCandidatus Contendobacter odensis PIE83306 methyltransferase Candidatus Contendobacter odensis
Candidatus Competibacter denitrificans WP 082161154 NR Bacteria Proteobacteria Gammaproteobacteria
0.4
Tree 1 - RQUA Group A dataset
Maximum-likelihood, IQTREE, UFBOOT - LG+C60
97 taxa, 206 sites

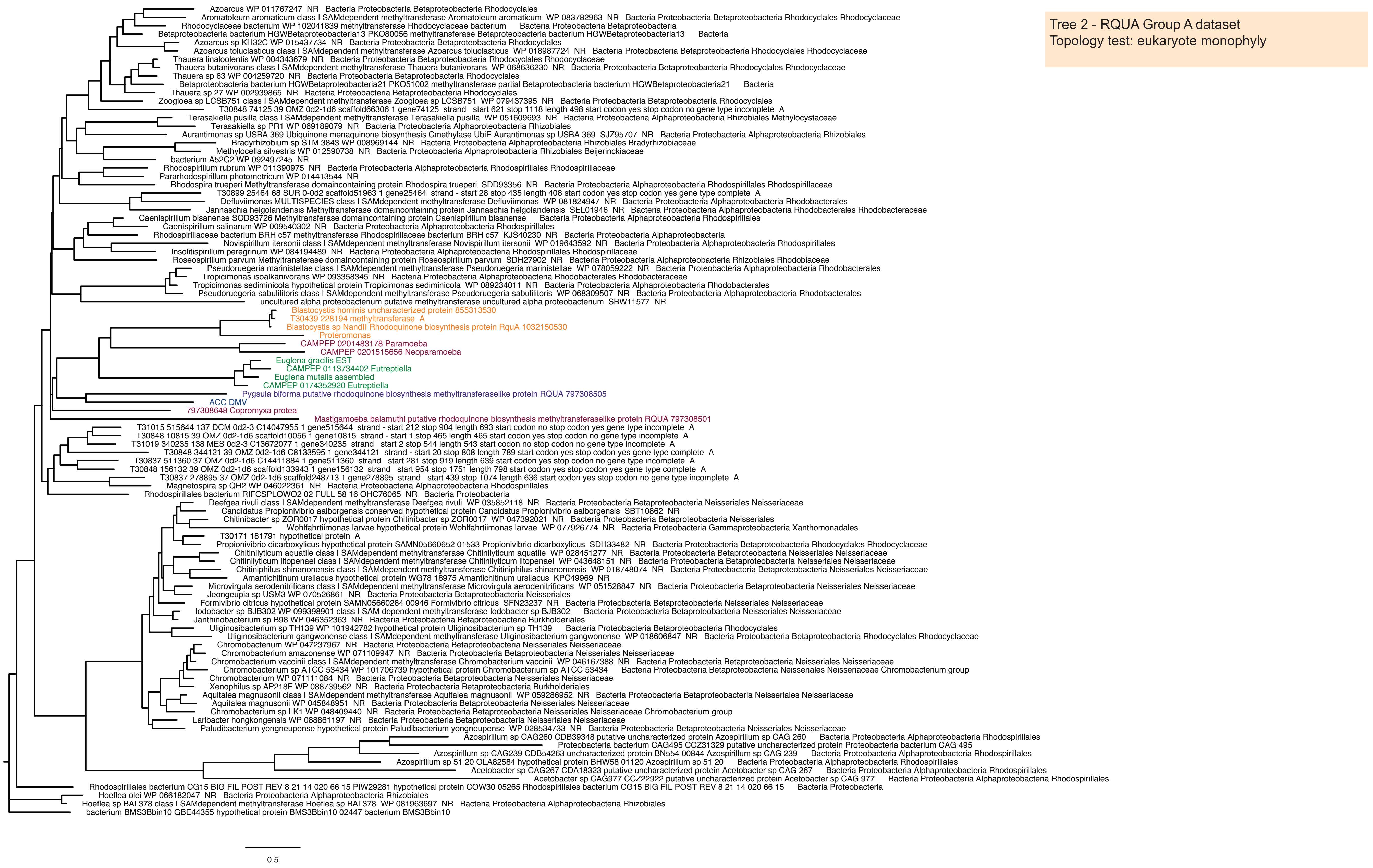
Tree 2 - RQUA Group A dataset
Topology test: eukaryote monophyly
Azoarcus WP 011767247 NR Bacteria Proteobacteria Betaproteobacteria Rhodocyclales
Aromatoleum aromaticum class I SAMdependent methyltransferase Aromatoleum aromaticum WP 083782963 NR Bacteria Proteobacteria Betaproteobacteria Rhodocyclales Rhodocyclaceae
Rhodocyclaceae bacterium WP 102041839 methyltransferase Rhodocyclaceae bacterium Bacteria Proteobacteria Betaproteobacteria
Betaproteobacteria bacterium HGWBetaproteobacteria13 PKO80056 methyltransferase Betaproteobacteria bacterium HGWBetaproteobacteria13 Bacteria
Azoarcus sp KH32C WP 015437734 NR Bacteria Proteobacteria Betaproteobacteria Rhodocyclales
Azoarcus toluclasticus class I SAMdependent methyltransferase Azoarcus toluclasticus WP 018987724 NR Bacteria Proteobacteria Betaproteobacteria Rhodocyclales Rhodocyclaceae
Thauera linaloolentis WP 004343679 NR Bacteria Proteobacteria Betaproteobacteria Rhodocyclales Rhodocyclaceae
Thauera butanivorans class I SAMdependent methyltransferase Thauera butanivorans WP 068636230 NR Bacteria Proteobacteria Betaproteobacteria Rhodocyclales Rhodocyclaceae
Thauera sp 63 WP 004259720 NR Bacteria Proteobacteria Betaproteobacteria Rhodocyclales
Betaproteobacteria bacterium HGWBetaproteobacteria21 PKO51002 methyltransferase partial Betaproteobacteria bacterium HGWBetaproteobacteria21 Bacteria
Thauera sp 27 WP 002939865 NR Bacteria Proteobacteria Betaproteobacteria Rhodocyclales
Zoogloea sp LCSB751 class I SAMdependent methyltransferase Zoogloea sp LCSB751 WP 079437395 NR Bacteria Proteobacteria Betaproteobacteria Rhodocyclales
T30848 74125 39 OMZ 0d2-1d6 scaffold66306 1 gene74125 strand start 621 stop 1118 length 498 start codon yes stop codon no gene type incomplete A
Terasakiella pusilla class I SAMdependent methyltransferase Terasakiella pusilla WP 015609693 NR Bacteria Proteobacteria Alphaproteobacteria Rhizobiales Methylocystaceae
Terasakiella sp PR1 WP 069189079 NR Bacteria Proteobacteria Alphaproteobacteria Rhizobiales
Aurantimonas sp USBA 369 Ubiquinone menaquinone biosynthesis Cmethylase UbiE Aurantimonas sp USBA 369 SJZ95707 NR Bacteria Proteobacteria Alphaproteobacteria Rhizobiales
Bradyrhizobium sp STM 3843 WP 008969144 NR Bacteria Proteobacteria Alphaproteobacteria Rhizobiales Bradyrhizobiaceae
Methylocella silvestris WP 012590738 NR Bacteria Proteobacteria Alphaproteobacteria Rhizobiales Beijerinckiaceae
bacterium A52C2 WP 092497245 NR
Rhodospirillum rubrum WP 011390975 NR Bacteria Proteobacteria Alphaproteobacteria Rhodospirillales Rhodospirillaceae
Pararhodospirillum photometricum WP 014413544 NR
Rhodospira trueperi Methyltransferase domaincontaining protein Rhodospira trueperi SDD93356 NR Bacteria Proteobacteria Alphaproteobacteria Rhodospirillales Rhodospirillaceae
T30899 25464 68 SUR 0-0d2 scaffold51963 1 gene25464 strand - start 28 stop 435 length 408 start codon yes stop codon yes gene type complete A
Defluviimonas MULTISPECIES class I SAMdependent methyltransferase Defluviimonas WP 081824947 NR Bacteria Proteobacteria Alphaproteobacteria Rhodobacterales
Jannaschia helgolandensis Methyltransferase domaincontaining protein Jannaschia helgolandensis SEL01946 NR Bacteria Proteobacteria Alphaproteobacteria Rhodobacterales Rhodobacteraceae
Caenispirillum bisanense SOD93726 Methyltransferase domaincontaining protein Caenispirillum bisanense Bacteria Proteobacteria Alphaproteobacteria Rhodospirillales
Caenispirillum salinarum WP 009540302 NR Bacteria Proteobacteria Alphaproteobacteria Rhodospirillales
Rhodospirillaceae bacterium BRH c57 methyltransferase Rhodospirillaceae bacterium BRH c57 KJS40230 NR Bacteria Proteobacteria Alphaproteobacteria
Novispirillum itersonii class I SAMdependent methyltransferase Novispirillum itersonii WP 019643592 NR Bacteria Proteobacteria Alphaproteobacteria Rhodospirillales
Insolitispirillum peregrinum WP 084194489 NR Bacteria Proteobacteria Alphaproteobacteria Rhodospirillales Rhodospirillaceae
Roseospirillum parvum Methyltransferase domaincontaining protein Roseospirillum parvum SDH27902 NR Bacteria Proteobacteria Alphaproteobacteria Rhizobiales Rhodobiaceae
Pseudoruegeria marinistellae class I SAMdependent methyltransferase Pseudoruegeria marinistellae WP 078059222 NR Bacteria Proteobacteria Alphaproteobacteria Rhodobacterales
Tropicimonas isoalkanivorans WP 093358345 NR Bacteria Proteobacteria Alphaproteobacteria Rhodobacterales Rhodobacteraceae
Tropicimonas sediminicola hypothetical protein Tropicimonas sediminicola WP 089234011 NR Bacteria Proteobacteria Alphaproteobacteria Rhodobacterales
Pseudoruegeria sabulilitoris class I SAMdependent methyltransferase Pseudoruegeria sabulilitoris WP 068309507 NR Bacteria Proteobacteria Alphaproteobacteria Rhodobacterales
uncultured alpha proteobacterium putative methyltransferase uncultured alpha proteobacterium SBW11577 NR
Blastocystis hominis uncharacterized protein 855313530
T30439 228194 methyltransferase A
Blastocystis sp NandII Rhodoquinone biosynthesis protein RquA 1032150530
Proteromonas
CAMPEP 0201483178 Paramoeba
CAMPEP 0201515656 Neoparamoeba
Euglena gracilis EST
CAMPEP 0113734402 Eutreptiella
Euglena mutalis assembled
CAMPEP 0174352920 Eutreptiella
Pygsuia biforma putative rhodoquinone biosynthesis methyltransferaselike protein RQUA 797308505
ACC DMV
797308648 Copromyxa protea
Mastigamoeba balamuthi putative rhodoquinone biosynthesis methyltransferaselike protein RQUA 797308501
T31015 515644 137 DCM 0d2-3 C14047955 1 gene515644 strand - start 212 stop 904 length 693 start codon no stop codon yes gene type incomplete A
T30848 10815 39 OMZ 0d2-1d6 scaffold10056 1 gene10815 strand - start 1 stop 465 length 465 start codon yes stop codon no gene type incomplete A
T31019 340235 138 MES 0d2-3 C13672077 1 gene340235 strand start 2 stop 544 length 543 start codon no stop codon no gene type incomplete A
T30848 344121 39 OMZ 0d2-1d6 C8133595 1 gene344121 strand - start 20 stop 808 length 789 start codon yes stop codon yes gene type complete A
T30837 511360 37 OMZ 0d2-1d6 C14411884 1 gene511360 strand start 281 stop 919 length 639 start codon yes stop codon no gene type incomplete A
T30848 156132 39 OMZ 0d2-1d6 scaffold133943 1 gene156132 strand start 954 stop 1751 length 798 start codon yes stop codon yes gene type complete A
T30837 278895 37 OMZ 0d2-1d6 scaffold248713 1 gene278895 strand start 439 stop 1074 length 636 start codon yes stop codon no gene type incomplete A
Magnetospira sp QH2 WP 046022361 NR Bacteria Proteobacteria Alphaproteobacteria Rhodospirillales
Rhodospirillales bacterium RIFCSPLOWO2 02 FULL 58 16 OHC76065 NR Bacteria Proteobacteria
Deefgea rivuli class I SAMdependent methyltransferase Deefgea rivuli WP 035852118 NR Bacteria Proteobacteria Betaproteobacteria Neisseriales Neisseriaceae
Candidatus Propionivibrio aalborgensis conserved hypothetical protein Candidatus Propionivibrio aalborgensis SBT10862 NR
Chitinibacter sp ZOR0017 hypothetical protein Chitinibacter sp ZOR0017 WP 047392021 NR Bacteria Proteobacteria Betaproteobacteria Neisseriales
Wohlfahrtiimonas larvae hypothetical protein Wohlfahrtiimonas larvae WP 077926774 NR Bacteria Proteobacteria Gammaproteobacteria Xanthomonadales
T30171 181791 hypothetical protein A
Propionivibrio dicarboxylicus hypothetical protein SAMN05660652 01533 Propionivibrio dicarboxylicus SDH33482 NR Bacteria Proteobacteria Betaproteobacteria Rhodocyclales Rhodocyclaceae
Chitinilyticum aquatile class I SAMdependent methyltransferase Chitinilyticum aquatile WP 028451277 NR Bacteria Proteobacteria Betaproteobacteria Neisseriales Neisseriaceae
Chitinilyticum litopenaei class I SAMdependent methyltransferase Chitinilyticum litopenaei WP 043648151 NR Bacteria Proteobacteria Betaproteobacteria Neisseriales Neisseriaceae
Chitiniphilus shinanonensis class I SAMdependent methyltransferase Chitiniphilus shinanonensis WP 018748074 NR Bacteria Proteobacteria Betaproteobacteria Neisseriales Neisseriaceae
Amantichitinum ursilacus hypothetical protein WG78 18975 Amantichitinum ursilacus KPC49969 NR
Microvirgula aerodenitrificans class I SAMdependent methyltransferase Microvirgula aerodenitrificans WP 051528847 NR Bacteria Proteobacteria Betaproteobacteria Neisseriales Neisseriaceae
Jeongeupia sp USM3 WP 070526861 NR Bacteria Proteobacteria Betaproteobacteria Neisseriales
Formivibrio citricus hypothetical protein SAMN05660284 00946 Formivibrio citricus SFN23237 NR Bacteria Proteobacteria Betaproteobacteria Neisseriales Neisseriaceae
Iodobacter sp BJB302 WP 099398901 class I SAM dependent methyltransferase Iodobacter sp BJB302 Bacteria Proteobacteria Betaproteobacteria Neisseriales Neisseriaceae
Janthinobacterium sp B98 WP 046352363 NR Bacteria Proteobacteria Betaproteobacteria Burkholderiales
Uliginosibacterium sp TH139 WP 101942782 hypothetical protein Uliginosibacterium sp TH139 Bacteria Proteobacteria Betaproteobacteria Rhodocyclales
Uliginosibacterium gangwonense class I SAMdependent methyltransferase Uliginosibacterium gangwonense WP 018606847 NR Bacteria Proteobacteria Betaproteobacteria Rhodocyclales Rhodocyclaceae
Chromobacterium WP 047237967 NR Bacteria Proteobacteria Betaproteobacteria Neisseriales Neisseriaceae
Chromobacterium amazonense WP 071109947 NR Bacteria Proteobacteria Betaproteobacteria Neisseriales Neisseriaceae
Chromobacterium vaccinii class I SAMdependent methyltransferase Chromobacterium vaccinii WP 046167388 NR Bacteria Proteobacteria Betaproteobacteria Neisseriales Neisseriaceae
Chromobacterium sp ATCC 53434 WP 101706739 hypothetical protein Chromobacterium sp ATCC 53434 Bacteria Proteobacteria Betaproteobacteria Neisseriales Neisseriaceae Chromobacterium group
Chromobacterium WP 071111084 NR Bacteria Proteobacteria Betaproteobacteria Neisseriales Neisseriaceae
Xenophilus sp AP218F WP 088739562 NR Bacteria Proteobacteria Betaproteobacteria Burkholderiales
Aquitalea magnusonii class I SAMdependent methyltransferase Aquitalea magnusonii WP 059286952 NR Bacteria Proteobacteria Betaproteobacteria Neisseriales Neisseriaceae
Aquitalea magnusonii WP 045848951 NR Bacteria Proteobacteria Betaproteobacteria Neisseriales Neisseriaceae
Chromobacterium sp LK1 WP 048409440 NR Bacteria Proteobacteria Betaproteobacteria Neisseriales Neisseriaceae Chromobacterium group
Laribacter hongkongensis WP 088861197 NR Bacteria Proteobacteria Betaproteobacteria Neisseriales Neisseriaceae
Paludibacterium yongneupense hypothetical protein Paludibacterium yongneupense WP 028534733 NR Bacteria Proteobacteria Betaproteobacteria Neisseriales Neisseriaceae
Azospirillum sp CAG260 CDB39348 putative uncharacterized protein Azospirillum sp CAG 260 Bacteria Proteobacteria Alphaproteobacteria Rhodospirillales
Proteobacteria bacterium CAG495 CCZ31329 putative uncharacterized protein Proteobacteria bacterium CAG 495
Azospirillum sp CAG239 CDB54263 uncharacterized protein BN554 00844 Azospirillum sp CAG 239 Bacteria Proteobacteria Alphaproteobacteria Rhodospirillales
Azospirillum sp 51 20 OLA82584 hypothetical protein BHW58 01120 Azospirillum sp 51 20 Bacteria Proteobacteria Alphaproteobacteria Rhodospirillales
Acetobacter sp CAG267 CDA18323 putative uncharacterized protein Acetobacter sp CAG 267 Bacteria Proteobacteria Alphaproteobacteria Rhodospirillales
Acetobacter sp CAG977 CCZ22922 putative uncharacterized protein Acetobacter sp CAG 977 Bacteria Proteobacteria Alphaproteobacteria Rhodospirillales
Rhodospirillales bacterium CG15 BIG FIL POST REV 8 21 14 020 66 15 PIW29281 hypothetical protein COW30 05265 Rhodospirillales bacterium CG15 BIG FIL POST REV 8 21 14 020 66 15 Bacteria Proteobacteria
Hoeflea olei WP 066182047 NR Bacteria Proteobacteria Alphaproteobacteria Rhizobiales
Hoeflea sp BAL378 class I SAMdependent methyltransferase Hoeflea sp BAL378 WP 081963697 NR Bacteria Proteobacteria Alphaproteobacteria Rhizobiales
bacterium BMS3Bbin10 GBE44355 hypothetical protein BMS3Bbin10 02447 bacterium BMS3Bbin10
0.5

Tree 3 - RQUA Group A dataset
Topology test: amoebozoa monophyly

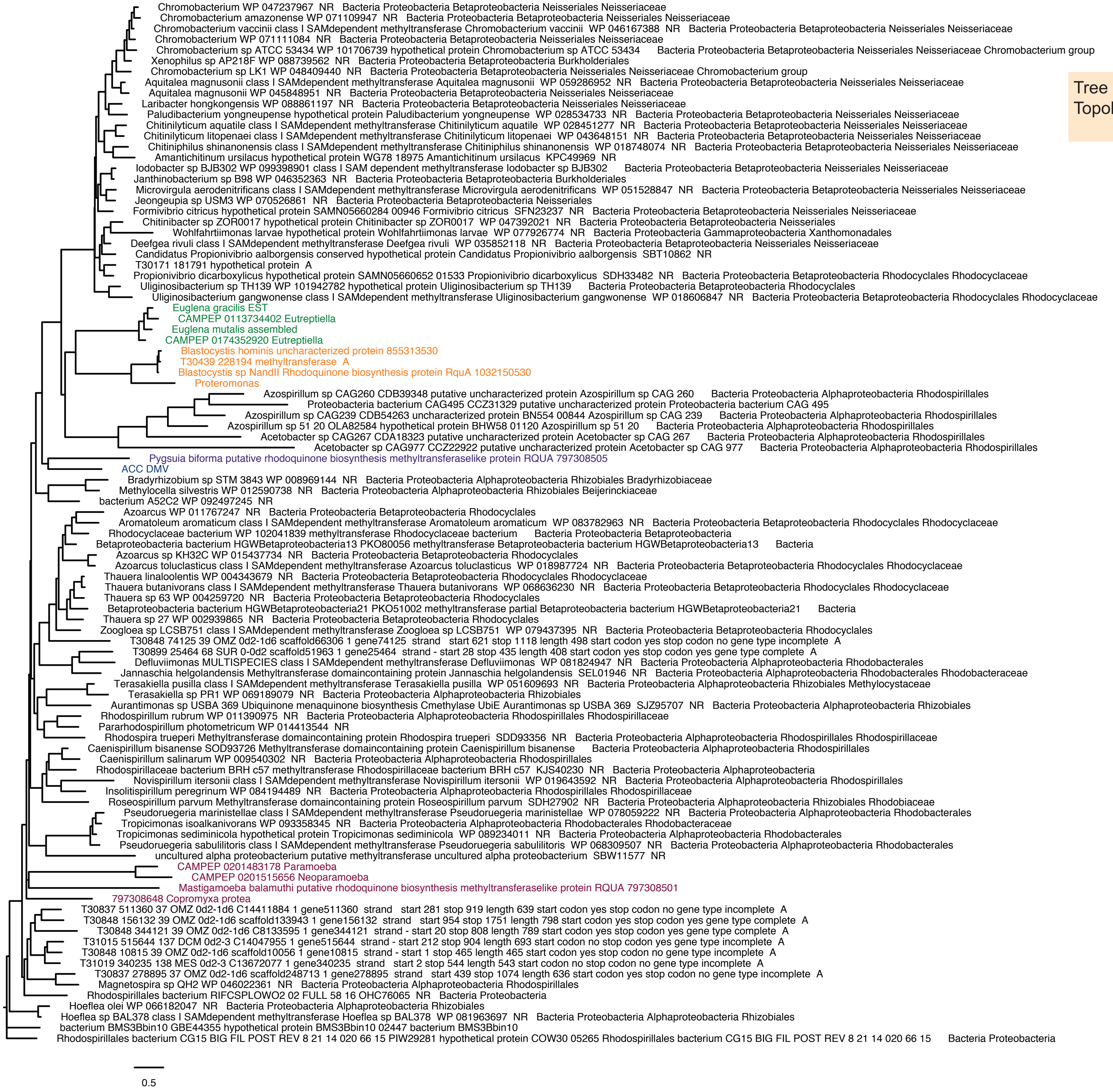

Tree 4 - RQUA Group A dataset
Topology test: amorphea monophyly

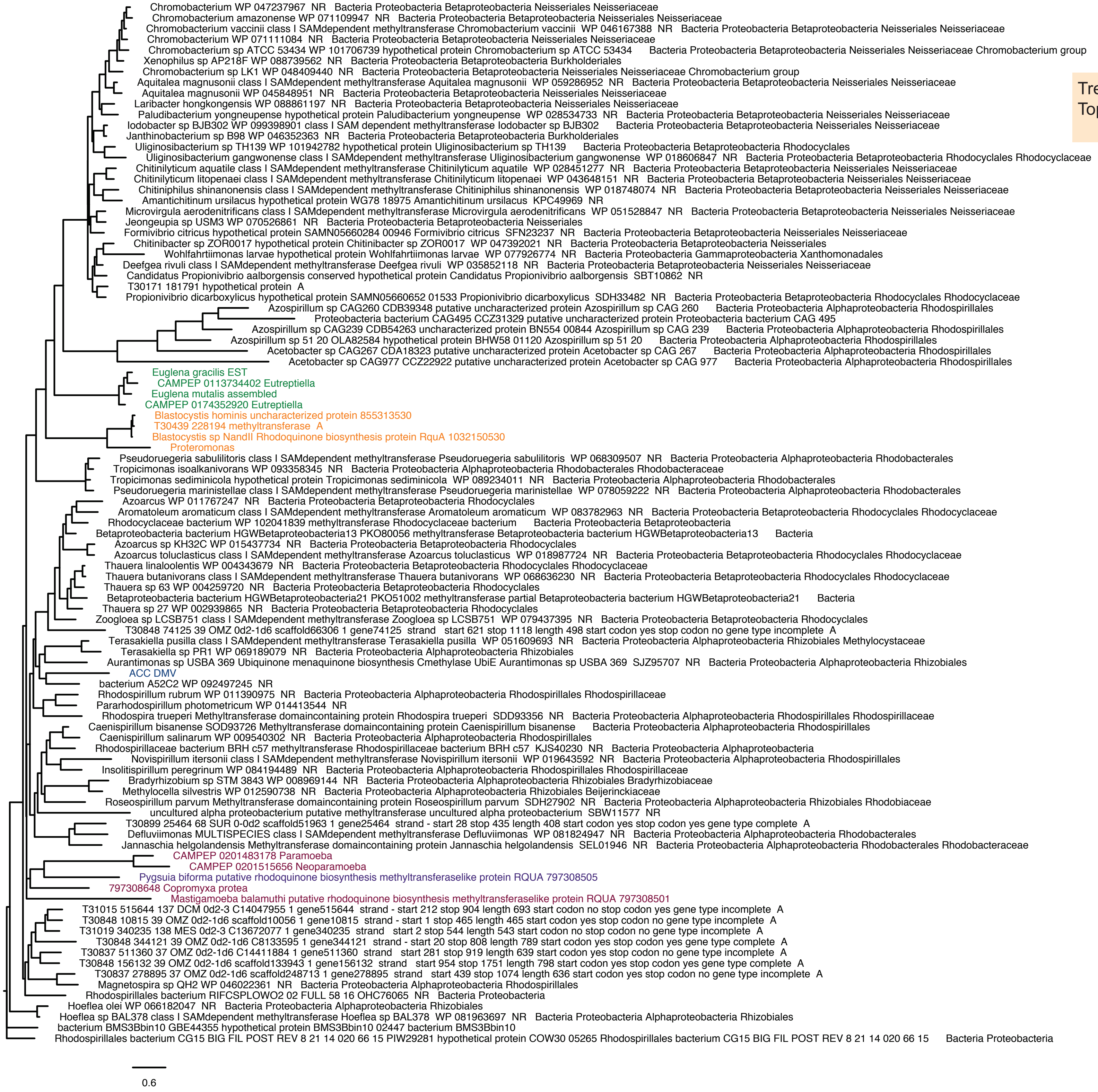

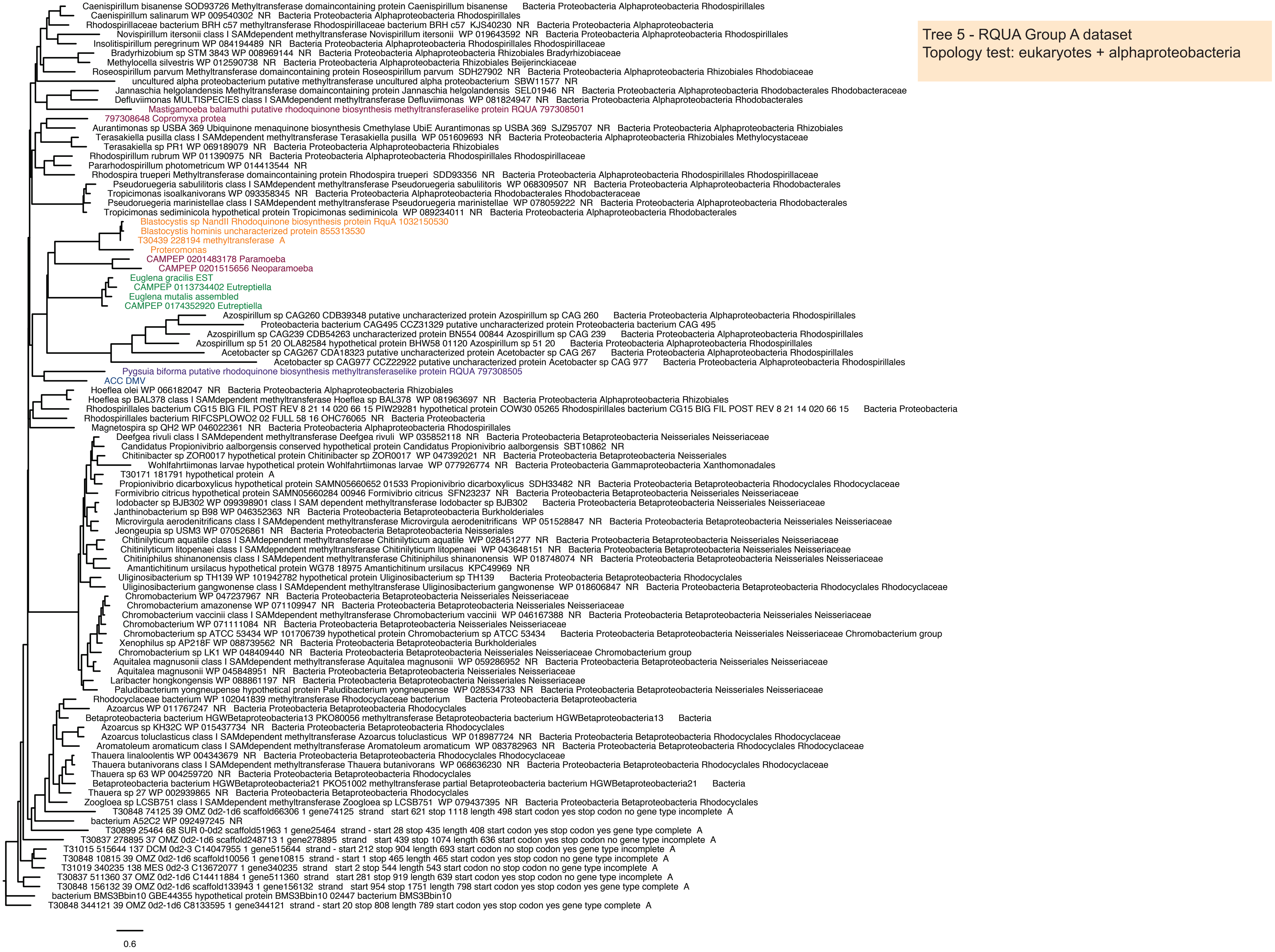
Tree 5 - RQUA Group A dataset
Topology test: eukaryotes + alphaproteobacteria
Caenispirillum bisanense SOD93726 Methyltransferase domaincontaining protein Caenispirillum bisanense Bacteria Proteobacteria Alphaproteobacteria Rhodospirillales
Caenispirillum salinarum WP 009540302 NR Bacteria Proteobacteria Alphaproteobacteria Rhodospirillales
Rhodospirillaceae bacterium BRH c57 methyltransferase Rhodospirillaceae bacterium BRH c57 KJS40230 NR Bacteria Proteobacteria Alphaproteobacteria
Novispirillum itersonii class I SAMdependent methyltransferase Novispirillum itersonii WP 019643592 NR Bacteria Proteobacteria Alphaproteobacteria Rhodospirillales
Insolitispirillum peregrinum WP 084194489 NR Bacteria Proteobacteria Alphaproteobacteria Rhodospirillales Rhodospirillaceae
Bradyrhizobium sp STM 3843 WP 008969144 NR Bacteria Proteobacteria Alphaproteobacteria Rhizobiales Bradyrhizobiaceae
Methylocella silvestris WP 012590738 NR Bacteria Proteobacteria Alphaproteobacteria Rhizobiales Beijerinckiaceae
Roseospirillum parvum Methyltransferase domaincontaining protein Roseospirillum parvum SDH27902 NR Bacteria Proteobacteria Alphaproteobacteria Rhizobiales Rhodobiaceae
uncultured alpha proteobacterium putative methyltransferase uncultured alpha proteobacterium SBW11577 NR
Jannaschia helgolandensis Methyltransferase domaincontaining protein Jannaschia helgolandensis SEL01946 NR Bacteria Proteobacteria Alphaproteobacteria Rhodobacterales Rhodobacteraceae
Defluviimonas MULTISPECIES class I SAMdependent methyltransferase Defluviimonas WP 081824947 NR Bacteria Proteobacteria Alphaproteobacteria Rhodobacterales
Mastigamoeba balamuthi putative rhodoquinone biosynthesis methyltransferaselike protein RQUA 797308501
797308648 Copromyxa protea
Aurantimonas sp USBA 369 Ubiquinone menaquinone biosynthesis Cmethylase UbiE Aurantimonas sp USBA 369 SJZ95707 NR Bacteria Proteobacteria Alphaproteobacteria Rhizobiales
Terasakiella pusilla class I SAMdependent methyltransferase Terasakiella pusilla WP 051609693 NR Bacteria Proteobacteria Alphaproteobacteria Rhizobiales Methylocystaceae
Terasakiella sp PR1 WP 069189079 NR Bacteria Proteobacteria Alphaproteobacteria Rhizobiales
Rhodospirillum rubrum WP 011390975 NR Bacteria Proteobacteria Alphaproteobacteria Rhodospirillales Rhodospirillaceae
Pararhodospirillum photometricum WP 014413544 NR
Rhodospira trueperi Methyltransferase domaincontaining protein Rhodospira trueperi SDD93356 NR Bacteria Proteobacteria Alphaproteobacteria Rhodospirillales Rhodospirillaceae
Pseudoruegeria sabulilitoris class I SAMdependent methyltransferase Pseudoruegeria sabulilitoris WP 068309507 NR Bacteria Proteobacteria Alphaproteobacteria Rhodobacterales
Tropicimonas isoalkanivorans WP 093358345 NR Bacteria Proteobacteria Alphaproteobacteria Rhodobacterales Rhodobacteraceae
Pseudoruegeria marinistellae class I SAMdependent methyltransferase Pseudoruegeria marinistellae WP 078059222 NR Bacteria Proteobacteria Alphaproteobacteria Rhodobacterales
Tropicimonas sediminicola hypothetical protein Tropicimonas sediminicola WP 089234011 NR Bacteria Proteobacteria Alphaproteobacteria Rhodobacterales
Blastocystis sp NandII Rhodoquinone biosynthesis protein RquA 1032150530
Blastocystis hominis uncharacterized protein 855313530
T30439 228194 methyltransferase A
Proteromonas
CAMPEP 0201483178 Paramoeba
CAMPEP 0201515656 Neoparamoeba
Euglena gracilis EST
CAMPEP 0113734402 Eutreptiella
Euglena mutalis assembled
CAMPEP 0174352920 Eutreptiella
Azospirillum sp CAG260 CDB39348 putative uncharacterized protein Azospirillum sp CAG 260 Bacteria Proteobacteria Alphaproteobacteria Rhodospirillales
Proteobacteria bacterium CAG495 CCZ31329 putative uncharacterized protein Proteobacteria bacterium CAG 495
Azospirillum sp CAG239 CDB54263 uncharacterized protein BN554 00844 Azospirillum sp CAG 239 Bacteria Proteobacteria Alphaproteobacteria Rhodospirillales
Azospirillum sp 51 20 OLA82584 hypothetical protein BHW58 01120 Azospirillum sp 51 20 Bacteria Proteobacteria Alphaproteobacteria Rhodospirillales
Acetobacter sp CAG267 CDA18323 putative uncharacterized protein Acetobacter sp CAG 267 Bacteria Proteobacteria Alphaproteobacteria Rhodospirillales
Acetobacter sp CAG977 CCZ22922 putative uncharacterized protein Acetobacter sp CAG 977 Bacteria Proteobacteria Alphaproteobacteria Rhodospirillales
Pygsuia biforma putative rhodoquinone biosynthesis methyltransferaselike protein RQUA 797308505
ACC DMV
Hoeflea olei WP 066182047 NR Bacteria Proteobacteria Alphaproteobacteria Rhizobiales
Hoeflea sp BAL378 class I SAMdependent methyltransferase Hoeflea sp BAL378 WP 081963697 NR Bacteria Proteobacteria Alphaproteobacteria Rhizobiales
Rhodospirillales bacterium CG15 BIG FIL POST REV 8 21 14 020 66 15 PIW29281 hypothetical protein COW30 05265 Rhodospirillales bacterium CG15 BIG FIL POST REV 8 21 14 020 66 15 Bacteria Proteobacteria
Rhodospirillales bacterium RIFCSPLOWO2 02 FULL 58 16 OHC76065 NR Bacteria Proteobacteria
Magnetospira sp QH2 WP 046022361 NR Bacteria Proteobacteria Alphaproteobacteria Rhodospirillales
Deefgea rivuli class I SAMdependent methyltransferase Deefgea rivuli WP 035852118 NR Bacteria Proteobacteria Betaproteobacteria Neisseriales Neisseriaceae
Candidatus Propionivibrio aalborgensis conserved hypothetical protein Candidatus Propionivibrio aalborgensis SBT10862 NR
Chitinibacter sp ZOR0017 hypothetical protein Chitinibacter sp ZOR0017 WP 047392021 NR Bacteria Proteobacteria Betaproteobacteria Neisseriales
Wohlfahrtiimonas larvae hypothetical protein Wohlfahrtiimonas larvae WP 077926774 NR Bacteria Proteobacteria Gammaproteobacteria Xanthomonadales
T30171 181791 hypothetical protein A
Propionivibrio dicarboxylicus hypothetical protein SAMN05660652 01533 Propionivibrio dicarboxylicus SDH33482 NR Bacteria Proteobacteria Betaproteobacteria Rhodocyclales Rhodocyclaceae
Formivibrio citricus hypothetical protein SAMN05660284 00946 Formivibrio citricus SFN23237 NR Bacteria Proteobacteria Betaproteobacteria Neisseriales Neisseriaceae
Iodobacter sp BJB302 WP 099398901 class I SAM dependent methyltransferase Iodobacter sp BJB302 Bacteria Proteobacteria Betaproteobacteria Neisseriales Neisseriaceae
Janthinobacterium sp B98 WP 046352363 NR Bacteria Proteobacteria Betaproteobacteria Burkholderiales
Microvirgula aerodenitrificans class I SAMdependent methyltransferase Microvirgula aerodenitrificans WP 051528847 NR Bacteria Proteobacteria Betaproteobacteria Neisseriales Neisseriaceae
Jeongeupia sp USM3 WP 070526861 NR Bacteria Proteobacteria Betaproteobacteria Neisseriales
Chitinilyticum aquatile class I SAMdependent methyltransferase Chitinilyticum aquatile WP 028451277 NR Bacteria Proteobacteria Betaproteobacteria Neisseriales Neisseriaceae
Chitinilyticum litopenaei class I SAMdependent methyltransferase Chitinilyticum litopenaei WP 043648151 NR Bacteria Proteobacteria Betaproteobacteria Neisseriales Neisseriaceae
Chitiniphilus shinanonensis class I SAMdependent methyltransferase Chitiniphilus shinanonensis WP 018748074 NR Bacteria Proteobacteria Betaproteobacteria Neisseriales Neisseriaceae
Amantichitinum ursilacus hypothetical protein WG78 18975 Amantichitinum ursilacus KPC49969 NR
Uliginosibacterium sp TH139 WP 101942782 hypothetical protein Uliginosibacterium sp TH139 Bacteria Proteobacteria Betaproteobacteria Rhodocyclales
Uliginosibacterium gangwonense class I SAMdependent methyltransferase Uliginosibacterium gangwonense WP 018606847 NR Bacteria Proteobacteria Betaproteobacteria Rhodocyclales Rhodocyclaceae
Chromobacterium WP 047237967 NR Bacteria Proteobacteria Betaproteobacteria Neisseriales Neisseriaceae
Chromobacterium amazonense WP 071109947 NR Bacteria Proteobacteria Betaproteobacteria Neisseriales Neisseriaceae
Chromobacterium vaccinii class I SAMdependent methyltransferase Chromobacterium vaccinii WP 046167388 NR Bacteria Proteobacteria Betaproteobacteria Neisseriales Neisseriaceae
Chromobacterium WP 071111084 NR Bacteria Proteobacteria Betaproteobacteria Neisseriales Neisseriaceae
Chromobacterium sp ATCC 53434 WP 101706739 hypothetical protein Chromobacterium sp ATCC 53434 Bacteria Proteobacteria Betaproteobacteria Neisseriales Neisseriaceae Chromobacterium group
Xenophilus sp AP218F WP 088739562 NR Bacteria Proteobacteria Betaproteobacteria Burkholderiales
Chromobacterium sp LK1 WP 048409440 NR Bacteria Proteobacteria Betaproteobacteria Neisseriales Neisseriaceae Chromobacterium group
Aquitalea magnusonii class I SAMdependent methyltransferase Aquitalea magnusonii WP 059286952 NR Bacteria Proteobacteria Betaproteobacteria Neisseriales Neisseriaceae
Aquitalea magnusonii WP 045848951 NR Bacteria Proteobacteria Betaproteobacteria Neisseriales Neisseriaceae
Laribacter hongkongensis WP 088861197 NR Bacteria Proteobacteria Betaproteobacteria Neisseriales Neisseriaceae
Paludibacterium yongneupense hypothetical protein Paludibacterium yongneupense WP 028534733 NR Bacteria Proteobacteria Betaproteobacteria Neisseriales Neisseriaceae
Rhodocyclaceae bacterium WP 102041839 methyltransferase Rhodocyclaceae bacterium Bacteria Proteobacteria Betaproteobacteria
Azoarcus WP 011767247 NR Bacteria Proteobacteria Betaproteobacteria Rhodocyclales
Betaproteobacteria bacterium HGWBetaproteobacteria13 PKO80056 methyltransferase Betaproteobacteria bacterium HGWBetaproteobacteria13 Bacteria
Azoarcus sp KH32C WP 015437734 NR Bacteria Proteobacteria Betaproteobacteria Rhodocyclales
Azoarcus toluclasticus class I SAMdependent methyltransferase Azoarcus toluclasticus WP 018987724 NR Bacteria Proteobacteria Betaproteobacteria Rhodocyclales Rhodocyclaceae
Aromatoleum aromaticum class I SAMdependent methyltransferase Aromatoleum aromaticum WP 083782963 NR Bacteria Proteobacteria Betaproteobacteria Rhodocyclales Rhodocyclaceae
Thauera linaloolentis WP 004343679 NR Bacteria Proteobacteria Betaproteobacteria Rhodocyclales Rhodocyclaceae
Thauera butanivorans class I SAMdependent methyltransferase Thauera butanivorans WP 068636230 NR Bacteria Proteobacteria Betaproteobacteria Rhodocyclales Rhodocyclaceae
Thauera sp 63 WP 004259720 NR Bacteria Proteobacteria Betaproteobacteria Rhodocyclales
Betaproteobacteria bacterium HGWBetaproteobacteria21 PKO51002 methyltransferase partial Betaproteobacteria bacterium HGWBetaproteobacteria21 Bacteria
Thauera sp 27 WP 002939865 NR Bacteria Proteobacteria Betaproteobacteria Rhodocyclales
Zoogloea sp LCSB751 class I SAMdependent methyltransferase Zoogloea sp LCSB751 WP 079437395 NR Bacteria Proteobacteria Betaproteobacteria Rhodocyclales
T30848 74125 39 OMZ 0d2-1d6 scaffold66306 1 gene74125 strand start 621 stop 1118 length 498 start codon yes stop codon no gene type incomplete A
bacterium A52C2 WP 092497245 NR
T30899 25464 68 SUR 0-0d2 scaffold51963 1 gene25464 strand - start 28 stop 435 length 408 start codon yes stop codon yes gene type complete A
T30837 278895 37 OMZ 0d2-1d6 scaffold248713 1 gene278895 strand start 439 stop 1074 length 636 start codon yes stop codon no gene type incomplete A
T31015 515644 137 DCM 0d2-3 C14047955 1 gene515644 strand - start 212 stop 904 length 693 start codon no stop codon yes gene type incomplete A
T30848 10815 39 OMZ 0d2-1d6 scaffold10056 1 gene10815 strand - start 1 stop 465 length 465 start codon yes stop codon no gene type incomplete A
T31019 340235 138 MES 0d2-3 C13672077 1 gene340235 strand start 2 stop 544 length 543 start codon no stop codon no gene type incomplete A
T30837 511360 37 OMZ 0d2-1d6 C14411884 1 gene511360 strand start 281 stop 919 length 639 start codon yes stop codon no gene type incomplete A
T30848 156132 39 OMZ 0d2-1d6 scaffold133943 1 gene156132 strand start 954 stop 1751 length 798 start codon yes stop codon yes gene type complete A
bacterium BMS3Bbin10 GBE44355 hypothetical protein BMS3Bbin10 02447 bacterium BMS3Bbin10
T30848 344121 39 OMZ 0d2-1d6 C8133595 1 gene344121 strand - start 20 stop 808 length 789 start codon yes stop codon yes gene type complete A
0.6

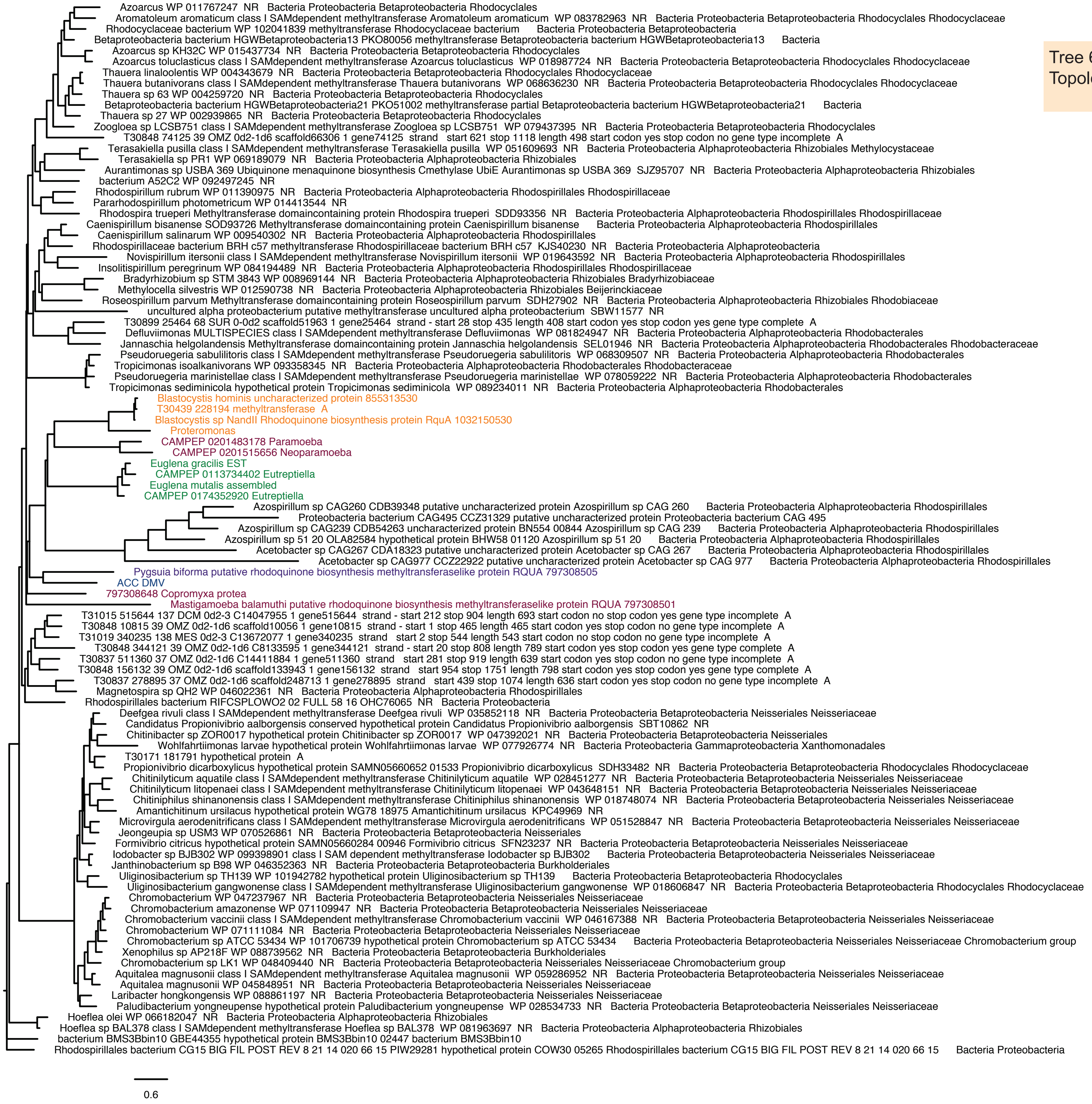

Tree 6 - RQUA Group A dataset
Topology test: eukaryotes + MAG alphaproteobacteria
