# Supplementary material for: Microbial eukaryotes have adapted to hypoxia by horizontal acquisitions of a gene involved in rhodoquinone biosynthesis
Source: eLife. 2018 Apr 26;7:e34292. doi: 10.7554/eLife.34292 (PMC5953543; doi:10.7554/eLife.34292)
Supplement: Supplementary file 4. [file elife-34292-supp4.pdf]

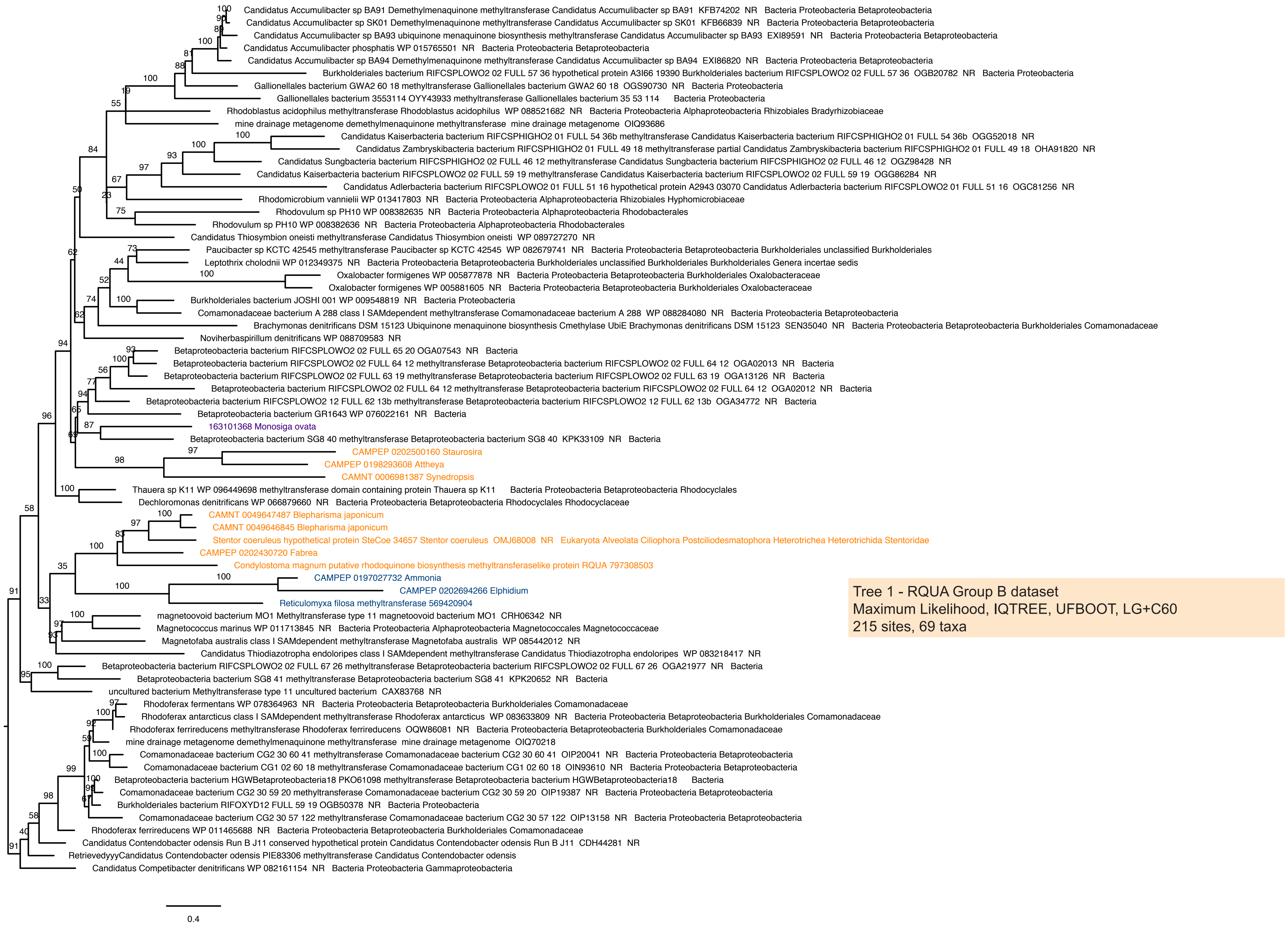
Candidatus Accumulibacter sp BA91 Demethylmenaquinone methyltransferase Candidatus Accumulibacter sp BA91 KFB74202 NR Bacteria Proteobacteria Betaproteobacteria
Candidatus Accumulibacter sp SK01 Demethylmenaquinone methyltransferase Candidatus Accumulibacter sp SK01 KFB66839 NR Bacteria Proteobacteria Betaproteobacteria
Candidatus Accumulibacter sp BA93 ubiquinone menaquinone biosynthesis methyltransferase Candidatus Accumulibacter sp BA93 EXI89591 NR Bacteria Proteobacteria Betaproteobacteria
Candidatus Accumulibacter phosphatis WP 015765501 NR Bacteria Proteobacteria Betaproteobacteria
Candidatus Accumulibacter sp BA94 Demethylmenaquinone methyltransferase Candidatus Accumulibacter sp BA94 EXI86820 NR Bacteria Proteobacteria Betaproteobacteria
Burkholderiales bacterium RIFCSPLOWO2 02 FULL 57 36 hypothetical protein A3I66 19390 Burkholderiales bacterium RIFCSPLOWO2 02 FULL 57 36 OGB20782 NR Bacteria Proteobacteria
Gallionellales bacterium GWA2 60 18 methyltransferase Gallionellales bacterium GWA2 60 18 OGS90730 NR Bacteria Proteobacteria
Gallionellales bacterium 3553114 OYY43933 methyltransferase Gallionellales bacterium 35 53 114 Bacteria Proteobacteria
Rhodoblastus acidophilus methyltransferase Rhodoblastus acidophilus WP 088521682 NR Bacteria Proteobacteria Alphaproteobacteria Rhizobiales Bradyrhizobiaceae
mine drainage metagenome demethylmenaquinone methyltransferase mine drainage metagenome OIQ93686
Candidatus Kaiserbacteria bacterium RIFCSPHIGHO2 01 FULL 54 36b methyltransferase Candidatus Kaiserbacteria bacterium RIFCSPHIGHO2 01 FULL 54 36b OGG52018 NR
Candidatus Zambryskibacteria bacterium RIFCSPHIGHO2 01 FULL 49 18 methyltransferase partial Candidatus Zambryskibacteria bacterium RIFCSPHIGHO2 01 FULL 49 18 OHA91820 NR
Candidatus Sungbacteria bacterium RIFCSPHIGHO2 02 FULL 46 12 methyltransferase Candidatus Sungbacteria bacterium RIFCSPHIGHO2 02 FULL 46 12 OGZ98428 NR
Candidatus Kaiserbacteria bacterium RIFCSPLOWO2 02 FULL 59 19 methyltransferase Candidatus Kaiserbacteria bacterium RIFCSPLOWO2 02 FULL 59 19 OGG86284 NR
Candidatus Adlerbacteria bacterium RIFCSPLOWO2 01 FULL 51 16 hypothetical protein A2943 03070 Candidatus Adlerbacteria bacterium RIFCSPLOWO2 01 FULL 51 16 OGC81256 NR
Rhodomicrobium vannielii WP 013417803 NR Bacteria Proteobacteria Alphaproteobacteria Rhizobiales Hyphomicrobiaceae
Rhodovulum sp PH10 WP 008382635 NR Bacteria Proteobacteria Alphaproteobacteria Rhodobacterales
Rhodovulum sp PH10 WP 008382636 NR Bacteria Proteobacteria Alphaproteobacteria Rhodobacterales
Candidatus Thiosymbion oneisti methyltransferase Candidatus Thiosymbion oneisti WP 089727270 NR
Paucibacter sp KCTC 42545 methyltransferase Paucibacter sp KCTC 42545 WP 082679741 NR Bacteria Proteobacteria Betaproteobacteria Burkholderiales unclassified Burkholderiales
Leptothrix cholodnii WP 012349375 NR Bacteria Proteobacteria Betaproteobacteria Burkholderiales unclassified Burkholderiales Burkholderiales Genera incertae sedis
Oxalobacter formigenes WP 005877878 NR Bacteria Proteobacteria Betaproteobacteria Burkholderiales Oxalobacteraceae
Oxalobacter formigenes WP 005881605 NR Bacteria Proteobacteria Betaproteobacteria Burkholderiales Oxalobacteraceae
Burkholderiales bacterium JOSHI 001 WP 009548819 NR Bacteria Proteobacteria
Comamonadaceae bacterium A 288 class I SAMdependent methyltransferase Comamonadaceae bacterium A 288 WP 088284080 NR Bacteria Proteobacteria Betaproteobacteria
Brachymonas denitrificans DSM 15123 Ubiquinone menaquinone biosynthesis Cmethylase UbiE Brachymonas denitrificans DSM 15123 SEN35040 NR Bacteria Proteobacteria Betaproteobacteria Burkholderiales Comamonadaceae
Noviherbaspirillum denitrificans WP 088709583 NR
Betaproteobacteria bacterium RIFCSPLOWO2 02 FULL 65 20 OGA07543 NR Bacteria
Betaproteobacteria bacterium RIFCSPLOWO2 02 FULL 64 12 methyltransferase Betaproteobacteria bacterium RIFCSPLOWO2 02 FULL 64 12 OGA02013 NR Bacteria
Betaproteobacteria bacterium RIFCSPLOWO2 02 FULL 63 19 methyltransferase Betaproteobacteria bacterium RIFCSPLOWO2 02 FULL 63 19 OGA13126 NR Bacteria
Betaproteobacteria bacterium RIFCSPLOWO2 02 FULL 64 12 methyltransferase Betaproteobacteria bacterium RIFCSPLOWO2 02 FULL 64 12 OGA02012 NR Bacteria
Betaproteobacteria bacterium RIFCSPLOWO2 12 FULL 62 13b methyltransferase Betaproteobacteria bacterium RIFCSPLOWO2 12 FULL 62 13b OGA34772 NR Bacteria
Betaproteobacteria bacterium GR1643 WP 076022161 NR Bacteria
163101368 Monosiga ovata
Betaproteobacteria bacterium SG8 40 methyltransferase Betaproteobacteria bacterium SG8 40 KPK33109 NR Bacteria
CAMPEP 0202500160 Staurosira
CAMPEP 0198293608 Attheya
CAMNT 0006981387 Synedropsis
Thauera sp K11 WP 096449698 methyltransferase domain containing protein Thauera sp K11 Bacteria Proteobacteria Betaproteobacteria Rhodocyclales
Dechloromonas denitrificans WP 066879660 NR Bacteria Proteobacteria Betaproteobacteria Rhodocyclales Rhodocyclaceae
CAMNT 0049647487 Blepharisma japonicum
CAMNT 0049646845 Blepharisma japonicum
Stentor coeruleus hypothetical protein SteCoe 34657 Stentor coeruleus OMJ68008 NR Eukaryota Alveolata Ciliophora Postciliodesmatophora Heterotrichea Heterotrichida Stentoridae
CAMPEP 0202430720 Fabrea
Condylostoma magnum putative rhodoquinone biosynthesis methyltransferaselike protein RQUA 797308503
CAMPEP 0197027732 Ammonia
CAMPEP 0202694266 Elphidium
Reticulomyxa filosa methyltransferase 569420904
magnetoovoid bacterium MO1 Methyltransferase type 11 magnetoovoid bacterium MO1 CRH06342 NR
Magnetococcus marinus WP 011713845 NR Bacteria Proteobacteria Alphaproteobacteria Magnetococcales Magnetococcaceae
Magnetofaba australis class I SAMdependent methyltransferase Magnetofaba australis WP 085442012 NR
Candidatus Thiodiazotropha endoloripes class I SAMdependent methyltransferase Candidatus Thiodiazotropha endoloripes WP 083218417 NR
Betaproteobacteria bacterium RIFCSPLOWO2 02 FULL 67 26 methyltransferase Betaproteobacteria bacterium RIFCSPLOWO2 02 FULL 67 26 OGA21977 NR Bacteria
Betaproteobacteria bacterium SG8 41 methyltransferase Betaproteobacteria bacterium SG8 41 KPK20652 NR Bacteria
uncultured bacterium Methyltransferase type 11 uncultured bacterium CAX83768 NR
Rhodoferax fermentans WP 078364963 NR Bacteria Proteobacteria Betaproteobacteria Burkholderiales Comamonadaceae
Rhodoferax antarcticus class I SAMdependent methyltransferase Rhodoferax antarcticus WP 083633809 NR Bacteria Proteobacteria Betaproteobacteria Burkholderiales Comamonadaceae
Rhodoferax ferrireducens methyltransferase Rhodoferax ferrireducens OQW86081 NR Bacteria Proteobacteria Betaproteobacteria Burkholderiales Comamonadaceae
mine drainage metagenome demethylmenaquinone methyltransferase mine drainage metagenome OIQ70218
Comamonadaceae bacterium CG2 30 60 41 methyltransferase Comamonadaceae bacterium CG2 30 60 41 OIP20041 NR Bacteria Proteobacteria Betaproteobacteria
Comamonadaceae bacterium CG1 02 60 18 methyltransferase Comamonadaceae bacterium CG1 02 60 18 OIN93610 NR Bacteria Proteobacteria Betaproteobacteria
Betaproteobacteria bacterium HGWBetaproteobacteria18 PKO61098 methyltransferase Betaproteobacteria bacterium HGWBetaproteobacteria18 Bacteria
Comamonadaceae bacterium CG2 30 59 20 methyltransferase Comamonadaceae bacterium CG2 30 59 20 OIP19387 NR Bacteria Proteobacteria Betaproteobacteria
Burkholderiales bacterium RIFOXYD12 FULL 59 19 OGB50378 NR Bacteria Proteobacteria
Comamonadaceae bacterium CG2 30 57 122 methyltransferase Comamonadaceae bacterium CG2 30 57 122 OIP13158 NR Bacteria Proteobacteria Betaproteobacteria
Rhodoferax ferrireducens WP 011465688 NR Bacteria Proteobacteria Betaproteobacteria Burkholderiales Comamonadaceae
Candidatus Contendobacter odensis Run B J11 conserved hypothetical protein Candidatus Contendobacter odensis Run B J11 CDH44281 NR
RetrievedyyyCandidatus Contendobacter odensis PIE83306 methyltransferase Candidatus Contendobacter odensis
Candidatus Competibacter denitrificans WP 082161154 NR Bacteria Proteobacteria Gammaproteobacteria
Tree 1 - RQUA Group B dataset
Maximum Likelihood, IQTREE, UFBOOT, LG+C60
215 sites, 69 taxa
0.4

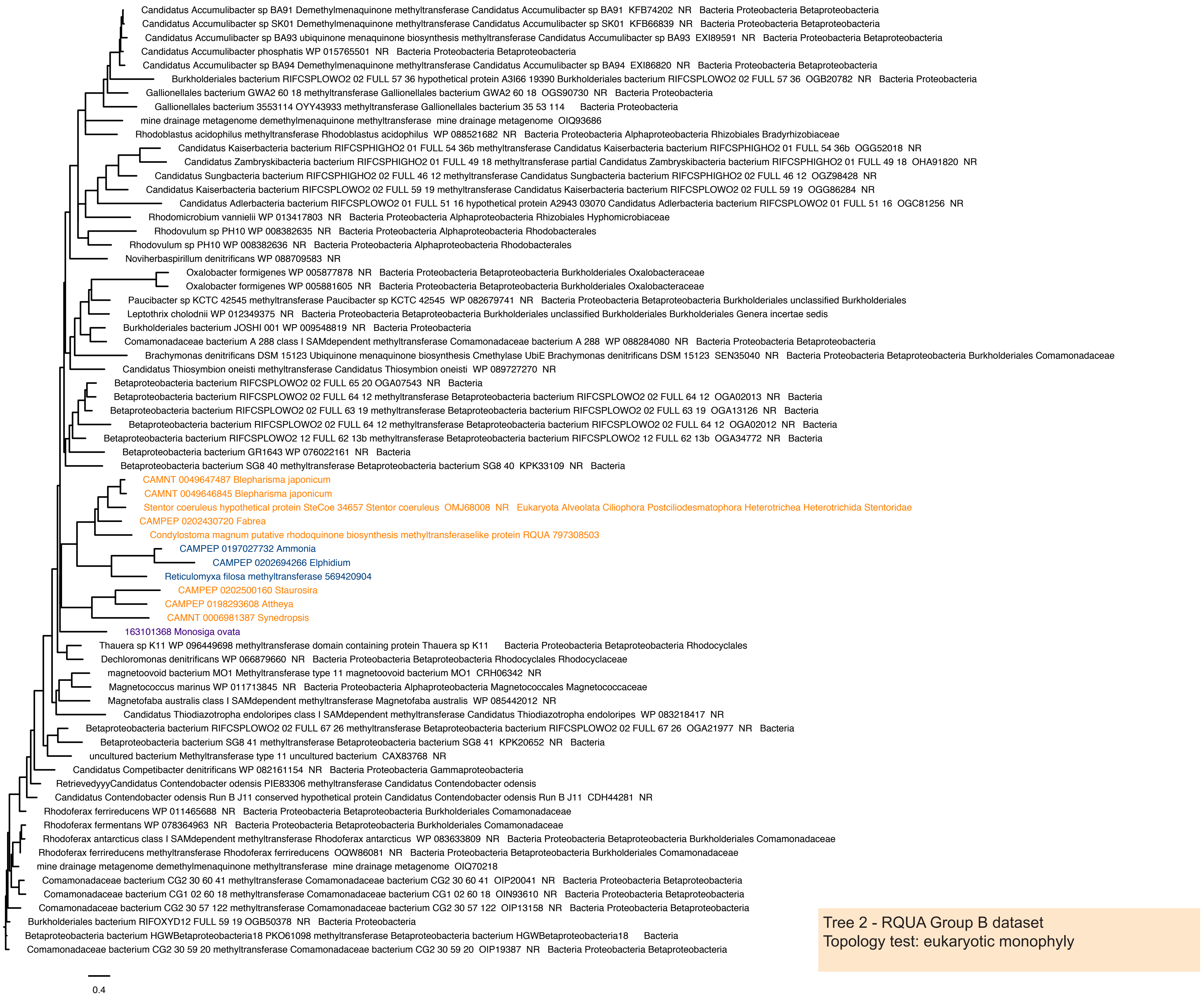
Candidatus Accumulibacter sp BA91 Demethylmenaquinone methyltransferase Candidatus Accumulibacter sp BA91 KFB74202 NR Bacteria Proteobacteria Betaproteobacteria
Candidatus Accumulibacter sp SK01 Demethylmenaquinone methyltransferase Candidatus Accumulibacter sp SK01 KFB66839 NR Bacteria Proteobacteria Betaproteobacteria
Candidatus Accumulibacter sp BA93 ubiquinone menaquinone biosynthesis methyltransferase Candidatus Accumulibacter sp BA93 EXI89591 NR Bacteria Proteobacteria Betaproteobacteria
Candidatus Accumulibacter phosphatis WP 015765501 NR Bacteria Proteobacteria Betaproteobacteria
Candidatus Accumulibacter sp BA94 Demethylmenaquinone methyltransferase Candidatus Accumulibacter sp BA94 EXI86820 NR Bacteria Proteobacteria Betaproteobacteria
Burkholderiales bacterium RIFCSPLOWO2 02 FULL 57 36 hypothetical protein A3I66 19390 Burkholderiales bacterium RIFCSPLOWO2 02 FULL 57 36 OGB20782 NR Bacteria Proteobacteria
Gallionellales bacterium GWA2 60 18 methyltransferase Gallionellales bacterium GWA2 60 18 OGS90730 NR Bacteria Proteobacteria
Gallionellales bacterium 3553114 OYY43933 methyltransferase Gallionellales bacterium 35 53 114 Bacteria Proteobacteria
mine drainage metagenome demethylmenaquinone methyltransferase mine drainage metagenome OIQ93686
Rhodoblastus acidophilus methyltransferase Rhodoblastus acidophilus WP 088521682 NR Bacteria Proteobacteria Alphaproteobacteria Rhizobiales Bradyrhizobiaceae
Candidatus Kaiserbacteria bacterium RIFCSPHIGHO2 01 FULL 54 36b methyltransferase Candidatus Kaiserbacteria bacterium RIFCSPHIGHO2 01 FULL 54 36b OGG52018 NR
Candidatus Zambryskibacteria bacterium RIFCSPHIGHO2 01 FULL 49 18 methyltransferase partial Candidatus Zambryskibacteria bacterium RIFCSPHIGHO2 01 FULL 49 18 OHA91820 NR
Candidatus Sungbacteria bacterium RIFCSPHIGHO2 02 FULL 46 12 methyltransferase Candidatus Sungbacteria bacterium RIFCSPHIGHO2 02 FULL 46 12 OGZ98428 NR
Candidatus Kaiserbacteria bacterium RIFCSPLOWO2 02 FULL 59 19 methyltransferase Candidatus Kaiserbacteria bacterium RIFCSPLOWO2 02 FULL 59 19 OGG86284 NR
Candidatus Adlerbacteria bacterium RIFCSPLOWO2 01 FULL 51 16 hypothetical protein A2943 03070 Candidatus Adlerbacteria bacterium RIFCSPLOWO2 01 FULL 51 16 OGC81256 NR
Rhodomicrobium vannielii WP 013417803 NR Bacteria Proteobacteria Alphaproteobacteria Rhizobiales Hyphomicrobiaceae
Rhodovulum sp PH10 WP 008382635 NR Bacteria Proteobacteria Alphaproteobacteria Rhodobacterales
Rhodovulum sp PH10 WP 008382636 NR Bacteria Proteobacteria Alphaproteobacteria Rhodobacterales
Noviherbaspirillum denitrificans WP 088709583 NR
Oxalobacter formigenes WP 005877878 NR Bacteria Proteobacteria Betaproteobacteria Burkholderiales Oxalobacteraceae
Oxalobacter formigenes WP 005881605 NR Bacteria Proteobacteria Betaproteobacteria Burkholderiales Oxalobacteraceae
Paucibacter sp KCTC 42545 methyltransferase Paucibacter sp KCTC 42545 WP 082679741 NR Bacteria Proteobacteria Betaproteobacteria Burkholderiales unclassified Burkholderiales
Leptothrix cholodnii WP 012349375 NR Bacteria Proteobacteria Betaproteobacteria Burkholderiales unclassified Burkholderiales Burkholderiales Genera incertae sedis
Burkholderiales bacterium JOSHI 001 WP 009548819 NR Bacteria Proteobacteria
Comamonadaceae bacterium A 288 class I SAMdependent methyltransferase Comamonadaceae bacterium A 288 WP 088284080 NR Bacteria Proteobacteria Betaproteobacteria
Brachymonas denitrificans DSM 15123 Ubiquinone menaquinone biosynthesis Cmethylase UbiE Brachymonas denitrificans DSM 15123 SEN35040 NR Bacteria Proteobacteria Betaproteobacteria Burkholderiales Comamonadaceae
Candidatus Thiosymbion oneisti methyltransferase Candidatus Thiosymbion oneisti WP 089727270 NR
Betaproteobacteria bacterium RIFCSPLOWO2 02 FULL 65 20 OGA07543 NR Bacteria
Betaproteobacteria bacterium RIFCSPLOWO2 02 FULL 64 12 methyltransferase Betaproteobacteria bacterium RIFCSPLOWO2 02 FULL 64 12 OGA02013 NR Bacteria
Betaproteobacteria bacterium RIFCSPLOWO2 02 FULL 63 19 methyltransferase Betaproteobacteria bacterium RIFCSPLOWO2 02 FULL 63 19 OGA13126 NR Bacteria
Betaproteobacteria bacterium RIFCSPLOWO2 02 FULL 64 12 methyltransferase Betaproteobacteria bacterium RIFCSPLOWO2 02 FULL 64 12 OGA02012 NR Bacteria
Betaproteobacteria bacterium RIFCSPLOWO2 12 FULL 62 13b methyltransferase Betaproteobacteria bacterium RIFCSPLOWO2 12 FULL 62 13b OGA34772 NR Bacteria
Betaproteobacteria bacterium GR1643 WP 076022161 NR Bacteria
Betaproteobacteria bacterium SG8 40 methyltransferase Betaproteobacteria bacterium SG8 40 KPK33109 NR Bacteria
CAMNT 0049647487 Blepharisma japonicum
CAMNT 0049646845 Blepharisma japonicum
Stentor coeruleus hypothetical protein SteCoe 34657 Stentor coeruleus OMJ68008 NR Eukaryota Alveolata Ciliophora Postciliodesmatophora Heterotrichea Heterotrichida Stentoridae
CAMPEP 0202430720 Fabrea
Condylostoma magnum putative rhodoquinone biosynthesis methyltransferaselike protein RQUA 797308503
CAMPEP 0197027732 Ammonia
CAMPEP 0202694266 Elphidium
Reticulomyxa filosa methyltransferase 569420904
CAMPEP 0202500160 Staurosira
CAMPEP 0198293608 Attheya
CAMNT 0006981387 Synedropsis
163101368 Monosiga ovata
Thauera sp K11 WP 096449698 methyltransferase domain containing protein Thauera sp K11 Bacteria Proteobacteria Betaproteobacteria Rhodocyclales
Dechloromonas denitrificans WP 066879660 NR Bacteria Proteobacteria Betaproteobacteria Rhodocyclales Rhodocyclaceae
magnetoovoid bacterium MO1 Methyltransferase type 11 magnetoovoid bacterium MO1 CRH06342 NR
Magnetococcus marinus WP 011713845 NR Bacteria Proteobacteria Alphaproteobacteria Magnetococcales Magnetococcaceae
Magnetofaba australis class I SAMdependent methyltransferase Magnetofaba australis WP 085442012 NR
Candidatus Thiodiazotropha endoloripes class I SAMdependent methyltransferase Candidatus Thiodiazotropha endoloripes WP 083218417 NR
Betaproteobacteria bacterium RIFCSPLOWO2 02 FULL 67 26 methyltransferase Betaproteobacteria bacterium RIFCSPLOWO2 02 FULL 67 26 OGA21977 NR Bacteria
Betaproteobacteria bacterium SG8 41 methyltransferase Betaproteobacteria bacterium SG8 41 KPK20652 NR Bacteria
uncultured bacterium Methyltransferase type 11 uncultured bacterium CAX83768 NR
Candidatus Competibacter denitrificans WP 082161154 NR Bacteria Proteobacteria Gammaproteobacteria
RetrievedyyyCandidatus Contendobacter odensis PIE83306 methyltransferase Candidatus Contendobacter odensis
Candidatus Contendobacter odensis Run B J11 conserved hypothetical protein Candidatus Contendobacter odensis Run B J11 CDH44281 NR
Rhodoferax ferrireducens WP 011465688 NR Bacteria Proteobacteria Betaproteobacteria Burkholderiales Comamonadaceae
Rhodoferax fermentans WP 078364963 NR Bacteria Proteobacteria Betaproteobacteria Burkholderiales Comamonadaceae
Rhodoferax antarcticus class I SAMdependent methyltransferase Rhodoferax antarcticus WP 083633809 NR Bacteria Proteobacteria Betaproteobacteria Burkholderiales Comamonadaceae
Rhodoferax ferrireducens methyltransferase Rhodoferax ferrireducens OQW86081 NR Bacteria Proteobacteria Betaproteobacteria Burkholderiales Comamonadaceae
mine drainage metagenome demethylmenaquinone methyltransferase mine drainage metagenome OIQ70218
Comamonadaceae bacterium CG2 30 60 41 methyltransferase Comamonadaceae bacterium CG2 30 60 41 OIP20041 NR Bacteria Proteobacteria Betaproteobacteria
Comamonadaceae bacterium CG1 02 60 18 methyltransferase Comamonadaceae bacterium CG1 02 60 18 OIN93610 NR Bacteria Proteobacteria Betaproteobacteria
Comamonadaceae bacterium CG2 30 57 122 methyltransferase Comamonadaceae bacterium CG2 30 57 122 OIP13158 NR Bacteria Proteobacteria Betaproteobacteria
Burkholderiales bacterium RIFOXYD12 FULL 59 19 OGB50378 NR Bacteria Proteobacteria
Betaproteobacteria bacterium HGWBetaproteobacteria18 PKO61098 methyltransferase Betaproteobacteria bacterium HGWBetaproteobacteria18 Bacteria
Comamonadaceae bacterium CG2 30 59 20 methyltransferase Comamonadaceae bacterium CG2 30 59 20 OIP19387 NR Bacteria Proteobacteria Betaproteobacteria
0.4
Tree 2 - RQUA Group B dataset
Topology test: eukaryotic monophyly

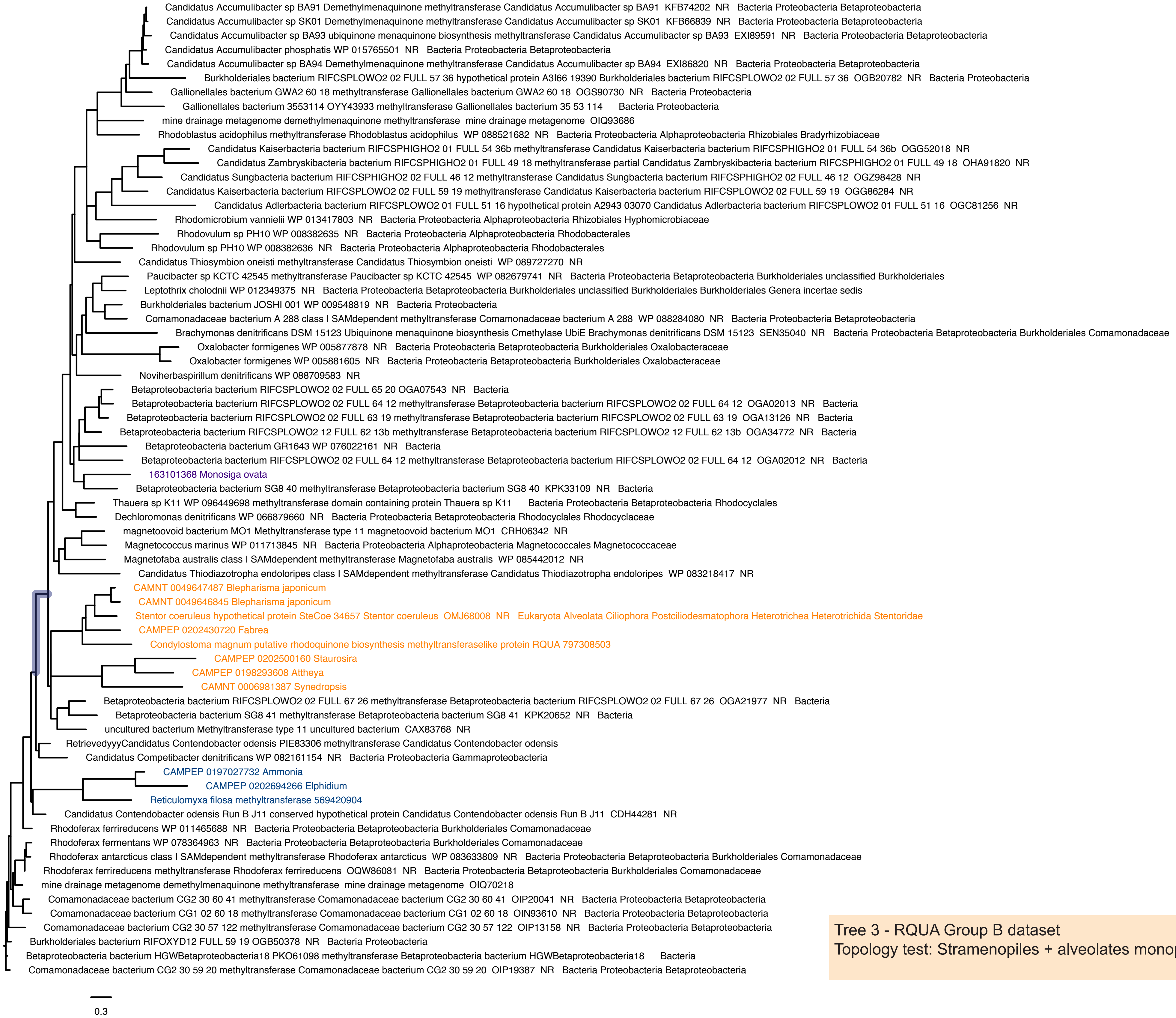

Tree 3 - RQUA Group B dataset
Topology test: Stramenopiles + alveolates monophyly

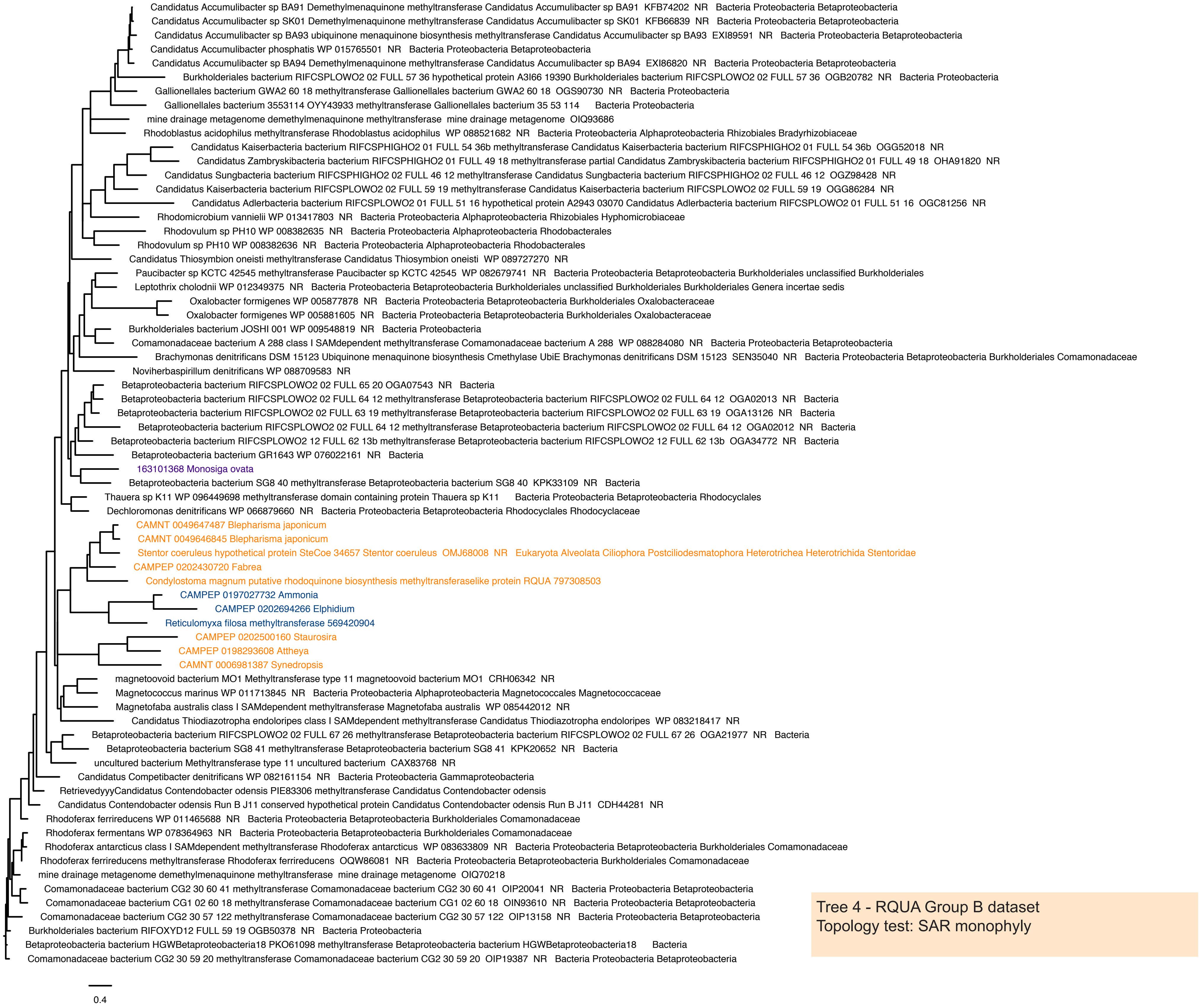
Candidatus Accumulibacter sp BA91 Demethylmenaquinone methyltransferase Candidatus Accumulibacter sp BA91 KFB74202 NR Bacteria Proteobacteria Betaproteobacteria
Candidatus Accumulibacter sp SK01 Demethylmenaquinone methyltransferase Candidatus Accumulibacter sp SK01 KFB66839 NR Bacteria Proteobacteria Betaproteobacteria
Candidatus Accumulibacter sp BA93 ubiquinone menaquinone biosynthesis methyltransferase Candidatus Accumulibacter sp BA93 EXI89591 NR Bacteria Proteobacteria Betaproteobacteria
Candidatus Accumulibacter phosphatis WP 015765501 NR Bacteria Proteobacteria Betaproteobacteria
Candidatus Accumulibacter sp BA94 Demethylmenaquinone methyltransferase Candidatus Accumulibacter sp BA94 EXI86820 NR Bacteria Proteobacteria Betaproteobacteria
Burkholderiales bacterium RIFCSPLOWO2 02 FULL 57 36 hypothetical protein A3I66 19390 Burkholderiales bacterium RIFCSPLOWO2 02 FULL 57 36 OGB20782 NR Bacteria Proteobacteria
Gallionellales bacterium GWA2 60 18 methyltransferase Gallionellales bacterium GWA2 60 18 OGS90730 NR Bacteria Proteobacteria
Gallionellales bacterium 3553114 OYY43933 methyltransferase Gallionellales bacterium 35 53 114 Bacteria Proteobacteria
mine drainage metagenome demethylmenaquinone methyltransferase mine drainage metagenome OIQ93686
Rhodoblastus acidophilus methyltransferase Rhodoblastus acidophilus WP 088521682 NR Bacteria Proteobacteria Alphaproteobacteria Rhizobiales Bradyrhizobiaceae
Candidatus Kaiserbacteria bacterium RIFCSPHIGHO2 01 FULL 54 36b methyltransferase Candidatus Kaiserbacteria bacterium RIFCSPHIGHO2 01 FULL 54 36b OGG52018 NR
Candidatus Zambryskibacteria bacterium RIFCSPHIGHO2 01 FULL 49 18 methyltransferase partial Candidatus Zambryskibacteria bacterium RIFCSPHIGHO2 01 FULL 49 18 OHA91820 NR
Candidatus Sungbacteria bacterium RIFCSPHIGHO2 02 FULL 46 12 methyltransferase Candidatus Sungbacteria bacterium RIFCSPHIGHO2 02 FULL 46 12 OGZ98428 NR
Candidatus Kaiserbacteria bacterium RIFCSPLOWO2 02 FULL 59 19 methyltransferase Candidatus Kaiserbacteria bacterium RIFCSPLOWO2 02 FULL 59 19 OGG86284 NR
Candidatus Adlerbacteria bacterium RIFCSPLOWO2 01 FULL 51 16 hypothetical protein A2943 03070 Candidatus Adlerbacteria bacterium RIFCSPLOWO2 01 FULL 51 16 OGC81256 NR
Rhodomicrobium vannielii WP 013417803 NR Bacteria Proteobacteria Alphaproteobacteria Rhizobiales Hyphomicrobiaceae
Rhodovulum sp PH10 WP 008382635 NR Bacteria Proteobacteria Alphaproteobacteria Rhodobacterales
Rhodovulum sp PH10 WP 008382636 NR Bacteria Proteobacteria Alphaproteobacteria Rhodobacterales
Candidatus Thiosymbion oneisti methyltransferase Candidatus Thiosymbion oneisti WP 089727270 NR
Paucibacter sp KCTC 42545 methyltransferase Paucibacter sp KCTC 42545 WP 082679741 NR Bacteria Proteobacteria Betaproteobacteria Burkholderiales unclassified Burkholderiales
Leptothrix cholodnii WP 012349375 NR Bacteria Proteobacteria Betaproteobacteria Burkholderiales unclassified Burkholderiales Burkholderiales Genera incertae sedis
Oxalobacter formigenes WP 005877878 NR Bacteria Proteobacteria Betaproteobacteria Burkholderiales Oxalobacteraceae
Oxalobacter formigenes WP 005881605 NR Bacteria Proteobacteria Betaproteobacteria Burkholderiales Oxalobacteraceae
Burkholderiales bacterium JOSHI 001 WP 009548819 NR Bacteria Proteobacteria
Comamonadaceae bacterium A 288 class I SAMdependent methyltransferase Comamonadaceae bacterium A 288 WP 088284080 NR Bacteria Proteobacteria Betaproteobacteria
Brachymonas denitrificans DSM 15123 Ubiquinone menaquinone biosynthesis Cmethylase UbiE Brachymonas denitrificans DSM 15123 SEN35040 NR Bacteria Proteobacteria Betaproteobacteria Burkholderiales Comamonadaceae
Noviherbaspirillum denitrificans WP 088709583 NR
Betaproteobacteria bacterium RIFCSPLOWO2 02 FULL 65 20 OGA07543 NR Bacteria
Betaproteobacteria bacterium RIFCSPLOWO2 02 FULL 64 12 methyltransferase Betaproteobacteria bacterium RIFCSPLOWO2 02 FULL 64 12 OGA02013 NR Bacteria
Betaproteobacteria bacterium RIFCSPLOWO2 02 FULL 63 19 methyltransferase Betaproteobacteria bacterium RIFCSPLOWO2 02 FULL 63 19 OGA13126 NR Bacteria
Betaproteobacteria bacterium RIFCSPLOWO2 02 FULL 64 12 methyltransferase Betaproteobacteria bacterium RIFCSPLOWO2 02 FULL 64 12 OGA02012 NR Bacteria
Betaproteobacteria bacterium RIFCSPLOWO2 12 FULL 62 13b methyltransferase Betaproteobacteria bacterium RIFCSPLOWO2 12 FULL 62 13b OGA34772 NR Bacteria
Betaproteobacteria bacterium GR1643 WP 076022161 NR Bacteria
163101368 Monosiga ovata
Betaproteobacteria bacterium SG8 40 methyltransferase Betaproteobacteria bacterium SG8 40 KPK33109 NR Bacteria
Thauera sp K11 WP 096449698 methyltransferase domain containing protein Thauera sp K11 Bacteria Proteobacteria Betaproteobacteria Rhodocyclales
Dechloromonas denitrificans WP 066879660 NR Bacteria Proteobacteria Betaproteobacteria Rhodocyclales Rhodocyclaceae
CAMNT 0049647487 Blepharisma japonicum
CAMNT 0049646845 Blepharisma japonicum
Stentor coeruleus hypothetical protein SteCoe 34657 Stentor coeruleus OMJ68008 NR Eukaryota Alveolata Ciliophora Postciliodesmatophora Heterotrichea Heterotrichida Stentoridae
CAMPEP 0202430720 Fabrea
Condylostoma magnum putative rhodoquinone biosynthesis methyltransferaselike protein RQUA 797308503
CAMPEP 0197027732 Ammonia
CAMPEP 0202694266 Elphidium
Reticulomyxa filosa methyltransferase 569420904
CAMPEP 0202500160 Staurosira
CAMPEP 0198293608 Attheya
CAMNT 0006981387 Synedropsis
magnetoovoid bacterium MO1 Methyltransferase type 11 magnetoovoid bacterium MO1 CRH06342 NR
Magnetococcus marinus WP 011713845 NR Bacteria Proteobacteria Alphaproteobacteria Magnetococcales Magnetococcaceae
Magnetofaba australis class I SAMdependent methyltransferase Magnetofaba australis WP 085442012 NR
Candidatus Thiodiazotropha endoloripes class I SAMdependent methyltransferase Candidatus Thiodiazotropha endoloripes WP 083218417 NR
Betaproteobacteria bacterium RIFCSPLOWO2 02 FULL 67 26 methyltransferase Betaproteobacteria bacterium RIFCSPLOWO2 02 FULL 67 26 OGA21977 NR Bacteria
Betaproteobacteria bacterium SG8 41 methyltransferase Betaproteobacteria bacterium SG8 41 KPK20652 NR Bacteria
uncultured bacterium Methyltransferase type 11 uncultured bacterium CAX83768 NR
Candidatus Competibacter denitrificans WP 082161154 NR Bacteria Proteobacteria Gammaproteobacteria
RetrievedyyyCandidatus Contendobacter odensis PIE83306 methyltransferase Candidatus Contendobacter odensis
Candidatus Contendobacter odensis Run B J11 conserved hypothetical protein Candidatus Contendobacter odensis Run B J11 CDH44281 NR
Rhodoferax ferrireducens WP 011465688 NR Bacteria Proteobacteria Betaproteobacteria Burkholderiales Comamonadaceae
Rhodoferax fermentans WP 078364963 NR Bacteria Proteobacteria Betaproteobacteria Burkholderiales Comamonadaceae
Rhodoferax antarcticus class I SAMdependent methyltransferase Rhodoferax antarcticus WP 083633809 NR Bacteria Proteobacteria Betaproteobacteria Burkholderiales Comamonadaceae
Rhodoferax ferrireducens methyltransferase Rhodoferax ferrireducens OQW86081 NR Bacteria Proteobacteria Betaproteobacteria Burkholderiales Comamonadaceae
mine drainage metagenome demethylmenaquinone methyltransferase mine drainage metagenome OIQ70218
Comamonadaceae bacterium CG2 30 60 41 methyltransferase Comamonadaceae bacterium CG2 30 60 41 OIP20041 NR Bacteria Proteobacteria Betaproteobacteria
Comamonadaceae bacterium CG1 02 60 18 methyltransferase Comamonadaceae bacterium CG1 02 60 18 OIN93610 NR Bacteria Proteobacteria Betaproteobacteria
Comamonadaceae bacterium CG2 30 57 122 methyltransferase Comamonadaceae bacterium CG2 30 57 122 OIP13158 NR Bacteria Proteobacteria Betaproteobacteria
Burkholderiales bacterium RIFOXYD12 FULL 59 19 OGB50378 NR Bacteria Proteobacteria
Betaproteobacteria bacterium HGWBetaproteobacteria18 PKO61098 methyltransferase Betaproteobacteria bacterium HGWBetaproteobacteria18 Bacteria
Comamonadaceae bacterium CG2 30 59 20 methyltransferase Comamonadaceae bacterium CG2 30 59 20 OIP19387 NR Bacteria Proteobacteria Betaproteobacteria
0.4
Tree 4 - RQUA Group B dataset
Topology test: SAR monophyly

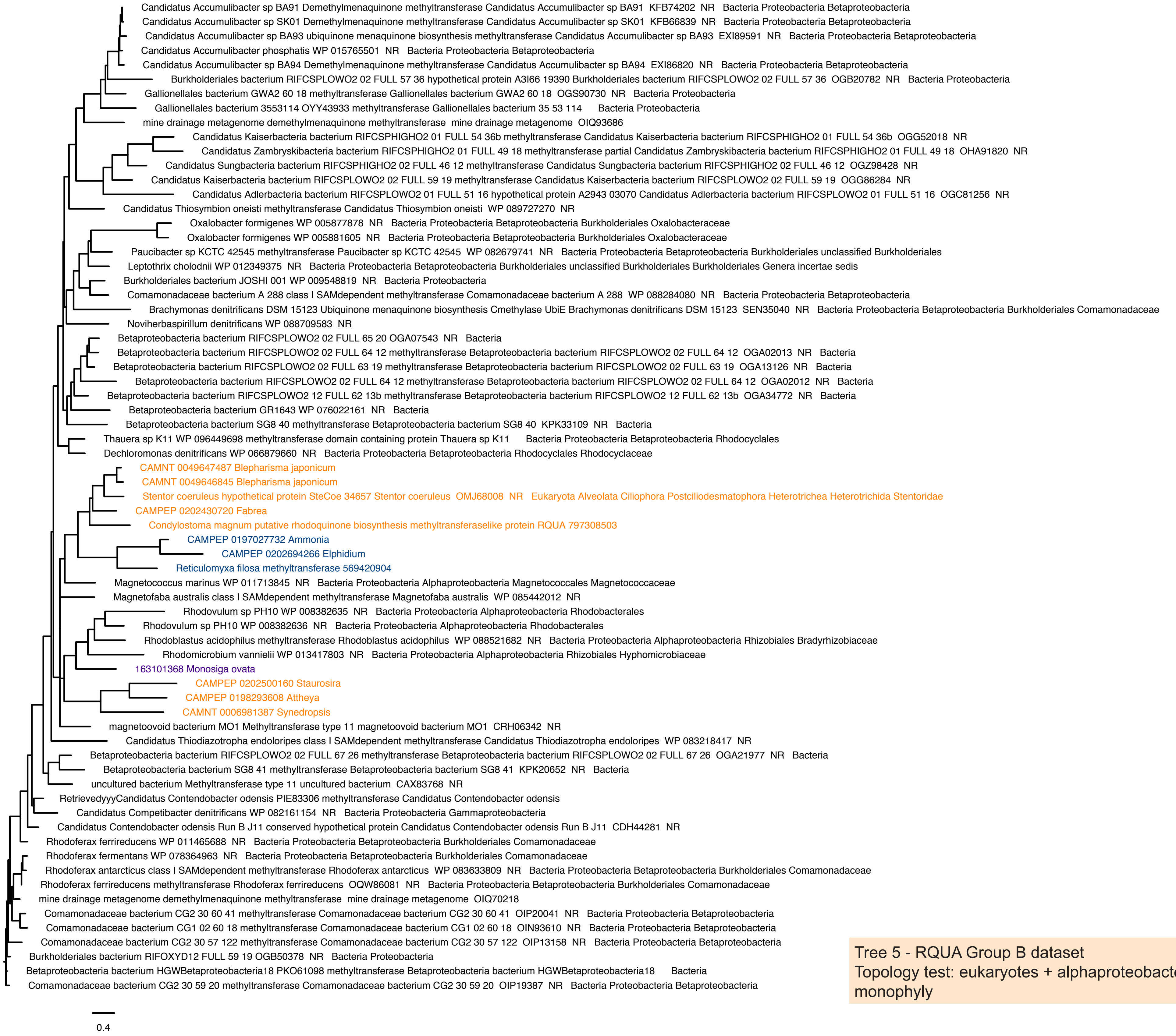

Tree 5 - RQUA Group B dataset
Topology test: eukaryotes + alphaproteobacteria monophyly
